# Sensing the Small Change of Intermolecular Distance in Supramolecular Assembly by Using the Tunable Emission Wavelength of AIE‐Active Luminogens

**DOI:** 10.1002/smll.202406511

**Published:** 2024-10-24

**Authors:** Xinyu Sun, Hui Li, Abdol Hadi Mokarizadeh, Xiaohan Xu, Tong Liu, Jiancheng Luo, Yuqing Yang, Shuailin Zhang, Fangbei Liu, Mesfin Tsige, Stephen Z. D. Cheng, Tianbo Liu

**Affiliations:** ^1^ School of Polymer Science and Polymer Engineering The University of Akron Akron OH 44325 USA

**Keywords:** aggregation‐induced emission (AIE), hybrid macromolecule, intermolecular distance indicator, supramolecular assembly

## Abstract

The distinct molecular states — single molecule, assembly, and aggregate — of two ionic macromolecules, TPPE‐APOSS and TPE‐APOSS, are easily distinguishable through their tunable fluorescence emission wavelengths, which reflect variations in intermolecular distances. Both ionic macromolecules contain aggregation‐induced emission (AIE) active moieties whose emission wavelengths are directly correlated to their mutual distances in solution: far away from each other as individual molecules, maintaining a tunable and relatively long distance in electrostatic interactions‐controlled blackberry‐type assemblies (microphase separation), or approaching van der Waals close distance in aggregates (macrophase separation). Furthermore, within the blackberry assemblies, the emission wavelength decreases monotonically with increasing assembly size, indicative of shorter intermolecular distances at nanoscale. The emission changes of TPPE‐APOSS blackberry assemblies can even be visually distinguishable by eyes when their sizes and intermolecular distances are tuned. Molecular dynamics simulations further revealed that macromolecules are confined in various conformations by controllable intermolecular distances within the blackberry structure, thereby resulting in fluorescence emission with tunable wavelength.

## Graphical abstract

The hybrid macromolecules incorporating AIE luminogens are designed to indicate the varying intermolecular distances in solutions through the tunable emission wavelength. They can sense not only distinct molecular states (discrete molecule, assembly, and aggregate) but also the nanoscale difference in the intermolecular distances within the electrostatic interaction‐controlled blackberry assemblies, indicated by the visually distinguishable fluorescence color change.

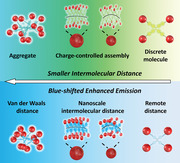

## 1 Introduction

Measuring intermolecular distance in non‐crystalline supramolecular assemblies is very challenging because the molecules often lack long‐range order and have a certain degree of freedom in molecular conformation and orientation. Using fluorescence signals to detect small changes in intermolecular distance is a promising approach if the signal can be directly correlated to the intermolecular distances. Fluorescent compounds have proven invaluable for probing the microenvironment, such as the existence of certain ions,^[^ ^1^, ^2^, ^3^ ^]^ chemicals,^[^ ^4^, ^5^, ^6^ ^]^ enantiomers,^[^ ^7^ ^]^ or the change of heat,^[^ ^8^ ^]^ force,^[^ ^9^, ^10^, ^11^ ^]^ pH,^[^ ^12^, ^13^ ^]^ etc. In recent studies, fluorescence has also been employed to study protein conformation changes and the formation of crystals of different forms.^[^ ^13^, ^14^, ^15^, ^16^ ^]^ However, direct correlation of fluorescence signals to intermolecular distances could be quite challenging as many fluorophores face aggregation‐caused quenching (ACQ) issues. But, if successful, fluorescence changes could offer a viable means to quantitively measure changes in intermolecular distances within assembly states.

Luminogens with the feature of aggregation‐induced emission (AIE) are good candidates to be applied to supramolecular structures.^[^ ^17^, ^18^, ^19^, ^20^, ^21^ ^]^ AIE luminogens are non‐emissive or weakly emissive in good solvents, while they emit strongly in the condensed phase.^[^ ^22^, ^23^, ^24^, ^25^ ^]^ The mechanism of the AIE luminance is the restriction of intramolecular motions (RIM), especially the restriction of intramolecular rotations (RIR), which blocks the non‐radiative relaxation channel and therefore opens the radiative pathway for AIE molecules to emit.^[^ ^26^ ^]^ Tetraphenylethylene (TPE) is one of the representative AIE lumingens, and tetra(biphenyl‐4‐yl) ethene (TPPE) is its derivative with extended conjugation.^[^ ^23^, ^25^, ^27^ ^]^ Both lumingens have been hybridized into metal‐organic cages (MOCs) and metal‐organic frameworks (MOFs),^[^ ^28^, ^29^, ^30^, ^31^, ^32^, ^33^ ^]^ exhibiting good sensitivity and tunable emission when undergoing environmental confinement.

Self‐assembly processes can involve multiple non‐covalent intramolecular interactions, such as hydrophobic interaction, electrostatic interaction, van der Waals forces, hydrogen bonding, and cation‐π interaction, etc. Each type of interaction has its own effective range and constrains the molecules to different extents. Macroscopic phase separation (common aggregation often based on van der Waals forces) or reversible amphiphilic assemblies (e.g., micelles, based on hydrophobic interaction) often leads to a well‐defined intermolecular distance in the assemblies where the molecules maintain a minimum van der Waals distance. On the other hand, long‐range electrostatic interaction can lead to assemblies with longer and tunable intermolecular distances. A wide range of nanosized macroions (1–5 nm), including polyoxometalates,^[^ ^34^ ^]^ polyhedral oligomeric silsesquioxane (POSS),^[^ ^35^ ^]^ functionalized fullerenes,^[^ ^36^ ^]^ and MOCs^[^ ^37^, ^38^ ^]^ have been reported to self‐assemble into single‐layered hollow spherical blackberry type structures through counterion‐mediated attraction in dilute solutions of polar solvents.^[^ ^39^, ^40^ ^]^ In contrast to the close intermolecular proximity fostered by hydrophobic interactions (van der Waals force), the macroions in the blackberry structures are distinctly separate and the blackberry membrane is permeable to small counterions.^[^ ^41^ ^]^ In that case, the molecules in the blackberry structure experience a different confinement environment compared to either the micelles and aggregates (restricted by van der Waals force) or soluble individual molecules (negligible restriction). Furthermore, the intermolecular distances in the blackberry assemblies are tunable through the control of macroion charge density, solvent polarity, pH, and ionic strength.^[^ ^42^, ^43^, ^44^, ^45^, ^46^ ^]^ Detecting the nanoscale differences in intermolecular distances is challenging yet highly motivated, and AIE luminogens present promising tools for this purpose.

**Scheme 1:**
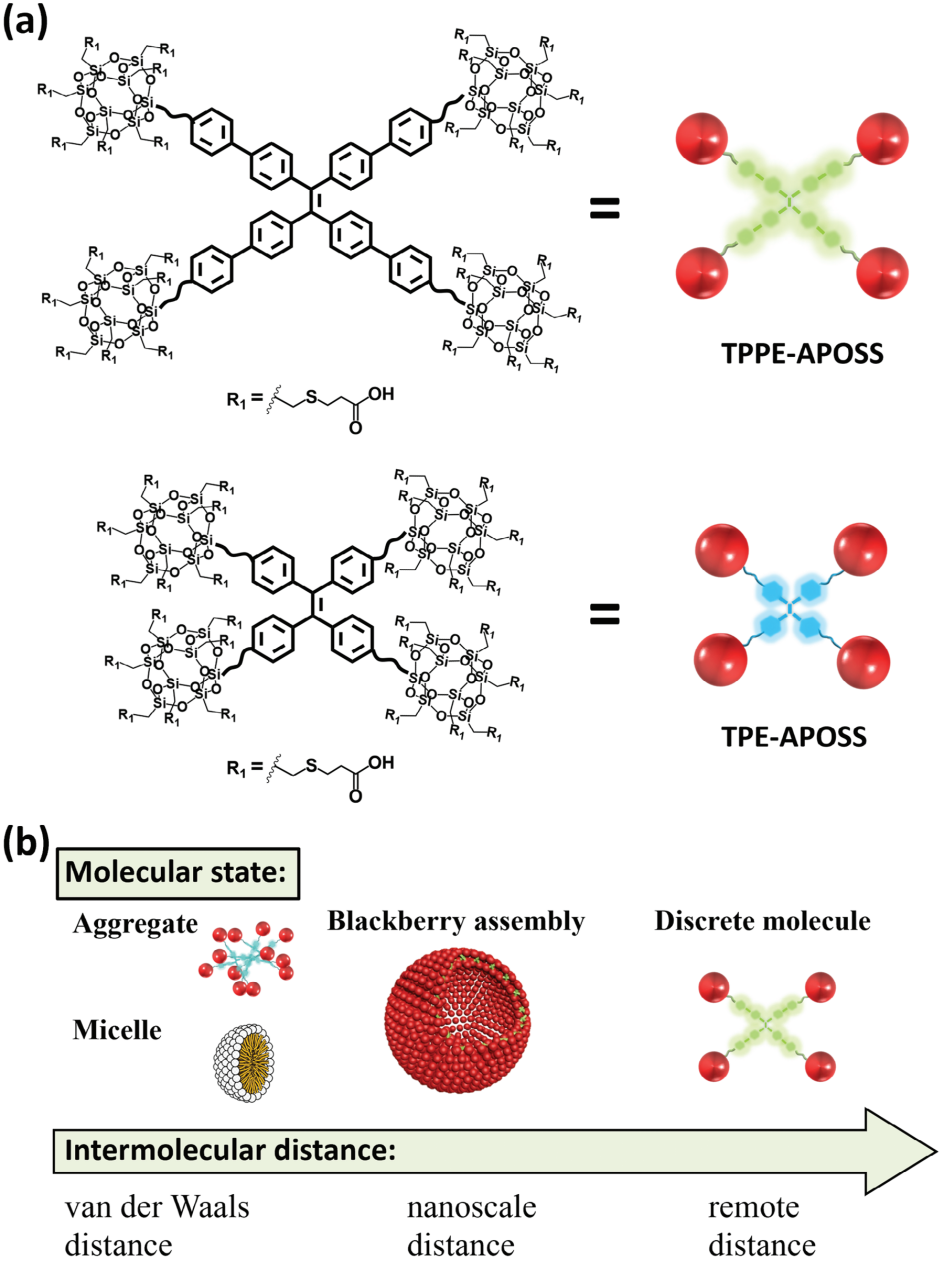
a) The molecular structures and cartoons of the AIE macromolecular hybrids **TPPE‐APOSS** and **TPE‐APOSS**. b) Illustration of intermolecular distances under varying molecular states.

A previous study of blackberry luminescence featuring TPPE based MOC successfully revealed the change of fluorescence color with varying intermolecular distances.^[^ ^30^ ^]^ However, MOCs encounter certain limitations as such indicators due to their minimal shift in emissive wavelength and susceptibility to solvents. To achieve more sensitive luminescence over a wider range, in this work, novel AIE macromolecules are synthesized by hybridizing TPPE or TPE luminogen with POSS based nanoclusters, creating four‐armed macromolecules TPPE‐APOSS and TPE‐APOSS (**Scheme** **1**). Such molecular design removes the framework restriction to the luminogen, which is expected to allow more conformation changes in these hybrids. The metal‐to‐ligand intramolecular charge transfer induced by the change in solvent polarity can also be avoided. Furthermore, these ionic macromolecules can be well regulated by electrostatic interaction, leading to tunable intermolecular distance (then tunable extents of motion restriction for the fluorophores and molecular conformations) in the blackberry assemblies. At last, the newly designed molecules avoid the intramolecular interaction between two TPPEs in the same MOC in the earlier work.^[^ ^47^ ^]^ These make them better indicators of intermolecular distances in assembly/aggregation states through direct visualization.

## 2 Results and Discussion

### 2.1 Molecular Design and Synthesis

**Figure 1:**
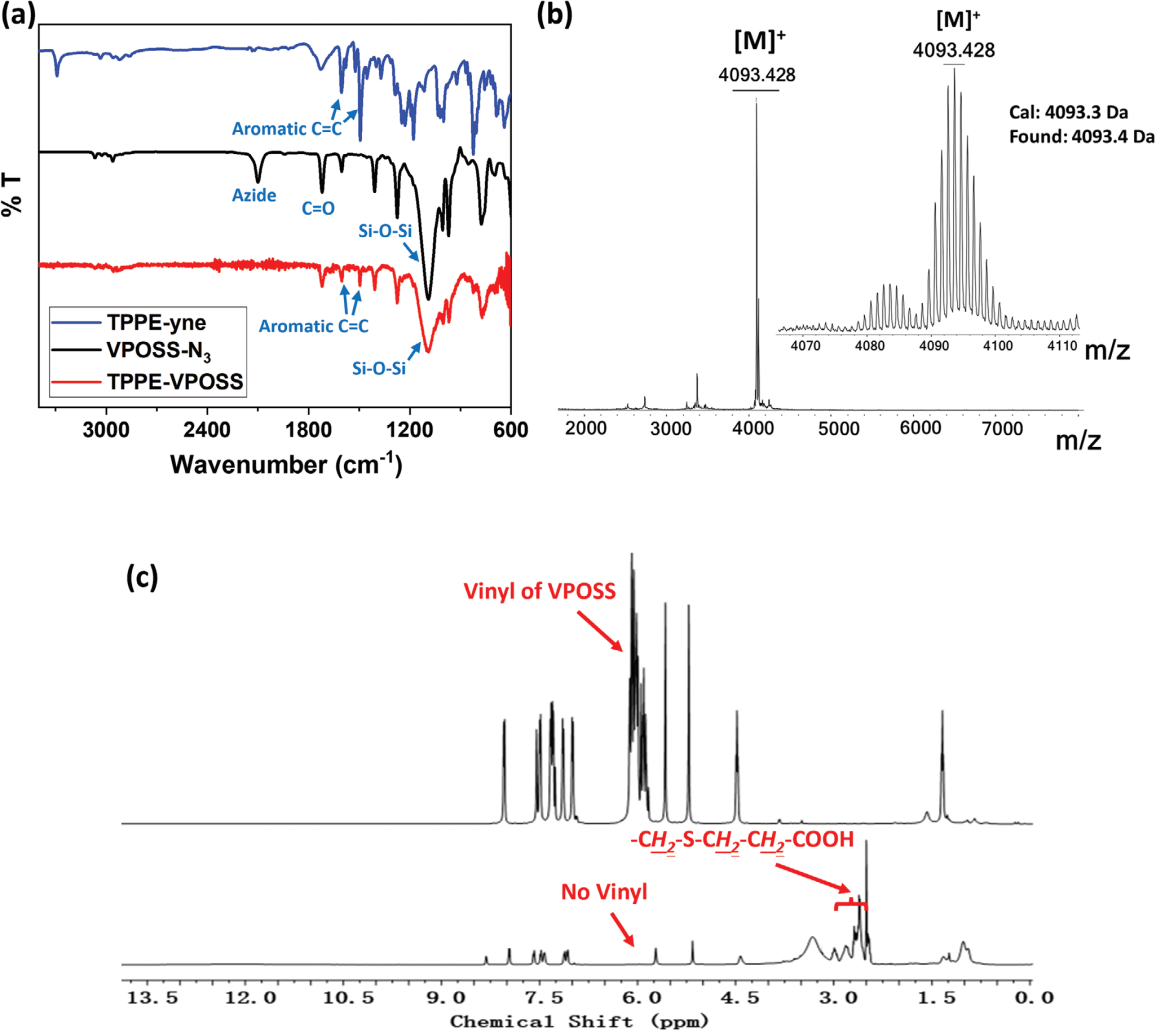
a) FT‐IR spectra of TPPE‐yne, VPOSS‐N_3_, and TPPE‐VPOSS. b) MALDI‐TOF spectrum of TPPE‐VPOSS. c) ^1^H (500 MHz) NMR spectra of TPPE‐APOSS in DMSO‐d_6_ (down).

The synthetic procedures to prepare the ionic TPPE‐APOSS macromolecules are described in the supporting information (Scheme S1, Supporting Information). TPPE‐yne was first synthesized from Tetrakis (4‐bromophenyl) ethylene through a sequence of reactions: Suzuki coupling reaction, demethylation, and nucleophilic substitution reaction (Figures S1–S4, Supporting Information). Subsequently, its four corners reacted with four VPOSS‐N_3_ via the copper(I)‐catalyzed alkyne−azide cycloaddition (CuAAC) “click” reaction to afford TPPE‐VPOSS. The azide groups from VPOSS‐N_3_ have strong absorption at 2100 cm^−1^ in FT‐IR spectrum, as shown in **Figure** **1**a. After the successful “click” reaction, this absorption of azide groups disappeared, while the characteristic Si─O─Si vibration band of the POSS cages at 1089 cm^−1^ remained. The characteristic peaks at 1604  and 1496 cm^−1^ (assigned to C═C vibration of phenyl rings in the TPPE core) demonstrated the successful introduction of TPPE moiety into TPPE‐VPOSS. The MALDI‐TOF mass spectrum of TPPE‐VPOSS in Figure 1b displayed the strongest peak of [M]^+^ located at *m/z* = 4093.4 Da, consistent with the calculated exact mass of 4093.3 Da. To functionalize TPPE‐VPOSS with hydrophilic carboxylic acid groups, converting it into TPPE‐APOSS, the thiol‐ene reaction with 3‐ mercaptopropionic acid was conducted under the illumination of 365 nm UV light. Monitored by ^1^H NMR, the signals of the vinyl groups of VPOSS (6.15–5.82 ppm) completely disappeared post‐reaction, while new peaks corresponding to the 3‐mercaptopropionic acid ligand appeared at 3.04–2.30 ppm, confirming the successful synthesis of TPPE‐APOSS (Figure 1c; Figures S5–S9, Supporting Information).

### 2.2 Optical Properties of Single TPPE‐VPOSS and TPPE‐APOSS Molecules in Solution

**Figure 2:**
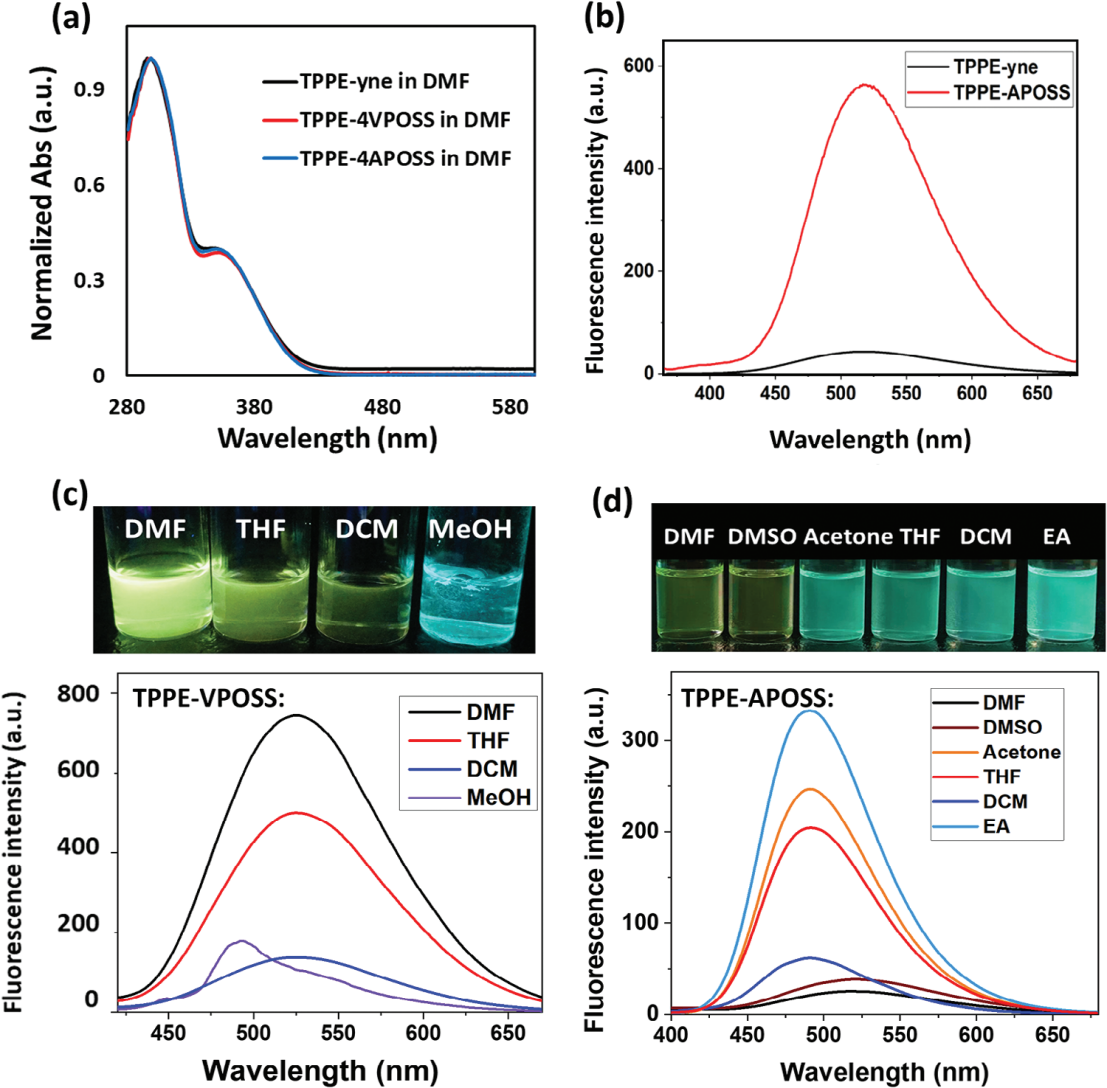
a) Normalized UV–vis spectra of TPPE‐yne, TPPE‐VPOSS, and TPPE‐APOSS in DMF. b) Fluorescence spectra of TPPE‐yne (2.9 mm) and TPPE‐APOSS (2.9 mm) in DMF. c,d) The photo (up) and spectra (down) of the fluorescence emission of 9.8 mm TPPE‐VPOSS (c) and 4.3 mm TPPE‐APOSS (d) in various solvents.

The UV–vis spectrum of the TPPE‐yne molecule displayed the absorption maxima at 300 and 350 nm when fully dissolved in DMF. The addition of four POSS units to the four TPPE‐yne corners does not alter the absorption peaks, indicating that the presence of POSS cages does not affect the π‐conjugation or the electronic interaction of the AIE luminogens (**Figure** **2**a). Therefore, the fluorescence of all the TPPE‐based compounds was excited at 350 nm for emission studies. When both TPPE‐yne and TPPE‐APOSS were dissolved in DMF at the same molar concentration as dilute solutions, the emission of TPPE‐yne was barely visible, while the yellow fluorescence emitted by TPPE‐APOSS was 10 times stronger (Figure 2b). This suggests that the existence of bulky POSS units induces a higher energy barrier to the rotation of C_Ph_─C_Ph_ single bonds, blocking the non‐radiative decay and therefore increases the fluorescence quantum yield from 0.26% to 3.05% (the quantum yields were calculated by using quinine sulfate as standard). The enhanced fluorescence of the single AIE molecule is usually observed in MOC and MOF due to the framework restrictions.^[^ ^29^, ^30^, ^33^, ^48^ ^]^ Here is the first observation of significant luminescence enhancement attributed to molecular branching.

The fluorescence emission of TPPE‐VPOSS and TPPE‐APOSS is sensitive to their solvation states, while not directly related to the solvent polarity. For example, dimethylformamide (DMF, ε_r_ = 36.7), tetrahydrofuran (THF, ε_r_ = 7.6), and dichloromethane (DCM, ε_r_ = 9.1) are good solvents for the nonpolar TPPE‐VPOSS, in which it can freely rotate. These solutions all emitted yellow fluorescence at the maximum wavelength of 520 nm, independent of solvent polarity (Figure 2c). Their UV–vis spectra also do not exhibit shift in absorption in response to solvent polarity (Figure S10, Supporting Information), indicating that TPPE‐VPOSS lacks charge separation or charge transfer induced by the solvent polarity. In poor solvents such as methanol which precipitates TPPE‐VPOSS, the suspension shows blue fluorescence (≈474 nm). In comparison, the surface charges of TPPE‐APOSS endow it with different solubility as well as the fluorescence behavior. In good solvents such as DMF and DMSO, the solutions are weakly emissive with the maximum wavelength still at 520 nm. However, the emission significantly enhances, and blue shifts to ≈491 nm (cyan) in poor solvents such as acetone, THF, DCM, and ethyl acetate (Figure 2d).

In addition to the typical feature of aggregation induced emission, a shift in the emissive wavelength was also observed from these macromolecules without the presence of internal charge transfer, photoinduced electron transfer, Förster resonance energy transfer, or metal‐to‐ligand charge‐transfer. Therefore, the shift in emissive wavelength should be attributed to the conjugation change through restricted molecular conformations. Previous studies on the TPPE conformation through single‐crystal analysis and the DFT calculations suggested that the blue‐shifted emission of TPPE derivates was due to the less electron overlap when TPPE twisted into larger dihedral angles in a confined framework.^[^ ^32^, ^48^, ^49^ ^]^ Therefore, suspension of TPPE‐VPOSS and TPPE‐APOSS aggregates both emitted at lower wavelengths probably because they were restricted in more twisted conformations.

### 2.3 Self‐Assembly of TPPE‐APOSS in DMF‐Acetone Mixture

**Figure 3:**
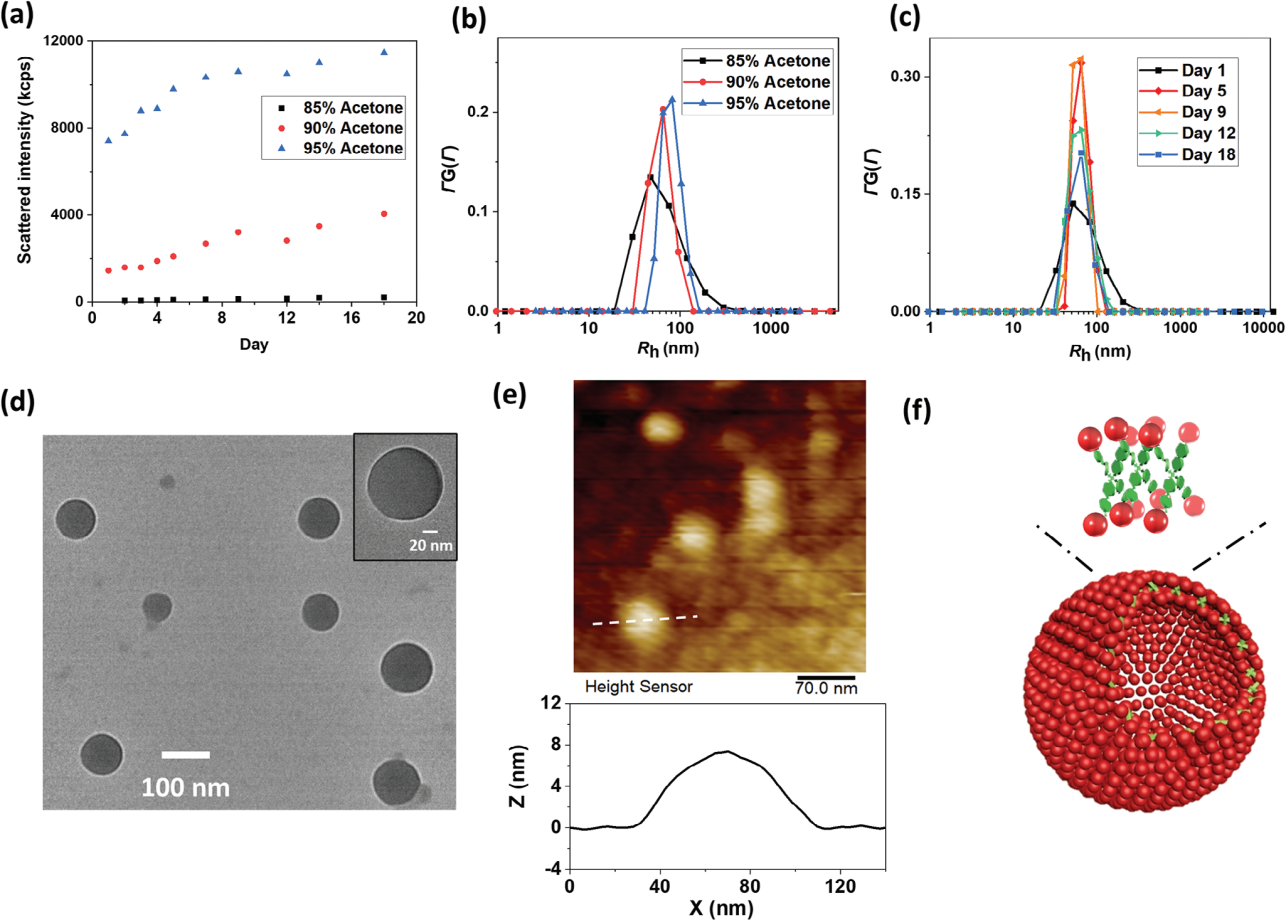
a) Light scattering intensity and b) *R*_h_ distributions of TPPE‐APOSS in DMF‐acetone mixture. c) *R*_h_ distributions of TPPE‐APOSS in 90 v/v% acetone/DMF over days. d) TEM imaging of the TPPE‐APOSS assemblies freeze‐dried from 90 v/v% acetone/DMF. e) AFM imaging (up), and the height profile along the dashed line (down) of the freeze‐dried TPPE‐APOSS assemblies. f) The model of blackberry structure constructed by TPPE‐APOSS.

TPPE‐APOSS solutions were studied by laser light scattering technique. Its good solubility in DMF was confirmed by the low scattered light intensity close to the solvent level (≈10 kcps). Comparing the vibrational absorption band of the ionized COO^−^ group and protonated ‐COOH group, the carboxylic groups of APOSS are moderately deprotonated at a degree of ≈70% in DMF (Figure S11, Supporting Information). Adding acetone into its DMF solution lowered the deprotonation degree of APOSS and reduced the repulsion between TPPE‐APOSS molecules. Time resolved static light scattering (SLS) measurements on the DMF‐acetone mixture of TPPE‐APOSS (0.1 mg mL^−1^) exhibit a continuous growth of scattered intensity when the acetone fraction was above 85%, as illustrated in **Figures** **3**a and S12a (Supporting Information), suggesting a continuous self‐assembly process of TPPE‐APOSS into supramolecular structures. The scattered intensity of the solutions grew for 10 days before slowing down and reaching a plateau. Meanwhile, the average hydrodynamic radii (*R*_h_) from dynamic light scattering (DLS) studies remained unchanged (Figure 3c), suggesting that the increment of intensity was due to a growing number of the assemblies rather than their sizes. In solutions with acetone fraction increased from 85% to 95%, the average *R*_h_ increased from 47 to 77 nm (Figure 3b and **Table** **1**). For example, in 90 v/v% acetone/DMF solution: the average *R*_h_ value is 62.2 nm without angular dependence (Figure S12b, Supporting Information), suggesting isotropic assemblies, e.g., spheres, in solution. The Zimm plot fitting^[^ ^50^ ^]^ of the SLS measurement of the assemblies indicates a comparable radius of gyration (*R*_g_) with *R*_h_ (*R*_g_ = 64.8 ± 0.3 nm_,_ Figure S12c (Supporting Information); *R*_g_ */ R*_h_ ∼1), suggesting that these assembly spheres are hollow. Morphology characterization via TEM imaging and the thickness measurement from the AFM image further confirmed these features (Figure 3d,e; Figure S13, Supporting Information). Particularly, the thickness of the collapsed spheres was measured to be 7.9 ± 0.4 nm, which means the membrane was ≈3.95 nm in thickness. This is close to the molecular length of TPPE‐APOSS, strongly suggesting the single‐layered hollow spherical blackberry structures (Figure 3f).

**Table 1:** Fluorescence emission of TPPE‐APOSS, TPPE‐VPOSS, and TPE‐APOSS in different molecular states.

Sample	Molecular state			
	Aggregates	Blackberry Assembly		Single molecule
	FL_Max_ [nm]	R_h_ [nm]	FL_Max_ [nm]	FL_Max_ [nm]
**TPPE‐APOSS**	≈491	47	508	520
		62	500	
		77	493	
**TPPE‐VPOSS**	≈474	N/A	N/A	520
**TPE‐APOSS**	≈462	47	473	Not visible

The formation of blackberry structures by macroions, e.g., polyoxometalates,^[^ ^40^ ^]^ POSS clusters,^[^ ^35^ ^]^ and their hybrids,^[^ ^51^, ^52^ ^]^ via counterion‐mediated attraction have been well reported. In this case, the deprotonated TPPE‐APOSS hybrid acts as a macroanion in DMF. By introducing less polar solvent acetone, the decreasing number of deprotonated protons reduced the repulsion between adjacent macromolecules, and the counterions (H^+^) functioned as bridge to bring the macromolecules into close distance. The tunable size of blackberry structure is a general feature, with larger size (smaller curvature) being favored by stronger counterion‐mediated attraction.^[^ ^40^, ^42^ ^]^ Solution containing a higher fraction of acetone lowers the charge density of TPPE‐APOSS, leading to reduced repulsion between neighboring molecules, resulting in a closer intermolecular distance and consequently larger blackberry sizes.

### 2.4 Fluorescence Emission of TPPE‐APOSS in DMF‐ Acetone Mixture

**Figure 4:**
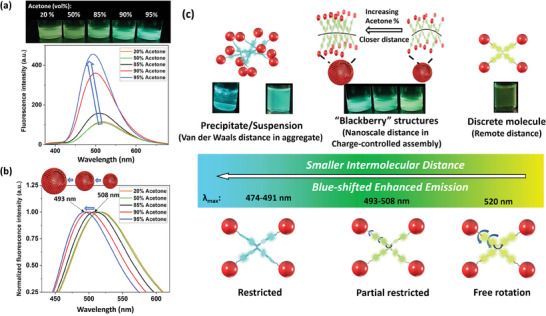
a) Photograph, fluorescence emission spectra, and b) normalized fluorescence emission spectra of TPPE‐APOSS in DMF‐acetone mixture with varying acetone fractions. c) The illustration of molecular states and their corresponding fluorescence emission.

The fluorescence spectra of the assemblies in DMF‐acetone mixture are shown in **Figure** **4**a and normalized in Figure 4b. At lower fractions of acetone when no assembly took place (e.g., <50% acetone), the solutions still exhibited weak yellow emission with maximum emission at ≈519 nm, similar to that of its pure DMF solution (∼520 nm), indicating that the macromolecules are in the single molecule state with the highest freedom of motion. With increasing acetone fraction, the solutions containing “blackberries” exhibited an obvious increase in emission (by 3.5‐fold in 95% acetone solution), accompanied by a gradual, monotonic shift toward the lower wavelength (508 nm–493 nm). Larger “blackberries” emitted at lower wavelengths, and the emissive wavelength shifted by 15 nm as the visible color changed from chartreuse to green and then cyan when the hydrodynamic radius (*R*_h_) of the blackberry structure increased from 47  to 77 nm. Detailed results are summarized in Table 1 and Figure 4c.

In TPPE‐APOSS solutions with higher acetone fractions, the macromolecules closer to each other within the blackberry structures. The increased emission intensity from solutions containing blackberries is due to the block of non‐radiative decay through rotational restriction by neighboring molecules in assemblies. The blue‐shifted fluorescence emission is due to the restriction molecular conformations to certain extents by varying intermolecular distances. This will be further illustrated through the simulation results later. The influence of other possibilities on the fluorescence wavelength changes such as the solvent polarity induced internal charge transfer has been excluded according to the discussion in the above text.

In addition, it is worth noting that the greenish emission from the blackberry solutions falls between the wavelengths of individual molecules (520 nm) and insoluble aggregates (491 nm). This implies that the motion of macromolecules is only partially restricted within the blackberry structures, allowing for some degree of C_ph_─C_ph_ single‐bond rotation and C═C double‐bond twist. This partial restriction on the molecular motion is in agreement with the feature of blackberry structures, where the distance between neighboring molecules, controlled by counterion‐mediated electrostatic attraction, is larger than that in aggregates attracted by van der Waals forces (<0.6 nm, or the primary minima).^[^ ^39^, ^43^, ^53^ ^]^ This also implies that the macromolecules arrange face‐to‐face on the blackberry surface, allowing the intermolecular distance to be regulated by the charge density of ionic macromolecules to provide different extents of partial rotational and vibrational freedom. The trends of the fluorescence response to molecular states and intermolecular distances are consistent with the observations from the TPPE‐based MOC,^[^ ^30^ ^]^ strongly indicating the universal feature of the AIE macroions. The fluorescence wavelength shift from TPPE‐APOSS “blackberries” is more significant and noticeable (15 nm) as compared to MOC “blackberries” (7 nm),^[^ ^30^ ^]^ attributed to the absence of framework restriction on the TPPE core. Taking advantage of the sensitive response of emission color to restricted conformations, this type of ionic AIE macromolecules have the potential to be used as an indicator for determining molecular states and more importantly the accurate intermolecular distance.

It's also worthwhile noticing that the suspension of neutral TPPE‐VPOSS aggregates emitted at a lower wavelength (474 nm, Figure 2c; Figure S14, Supporting Information) compared to ionic TPPE‐APOSS. This corresponds to the state where the insoluble molecules aggregate into van der Waals distance, which is shorter than the intermolecular distance in the charge‐controlled assembly state, leading to a higher extent of confinement. This emission wavelength can be considered the shortest possible for TPPE‐VPOSS molecules.

### 2.5 Self‐Assembly and Fluorescence Emission of TPE‐APOSS in DMF‐Acetone Mixture

**Figure 5:**
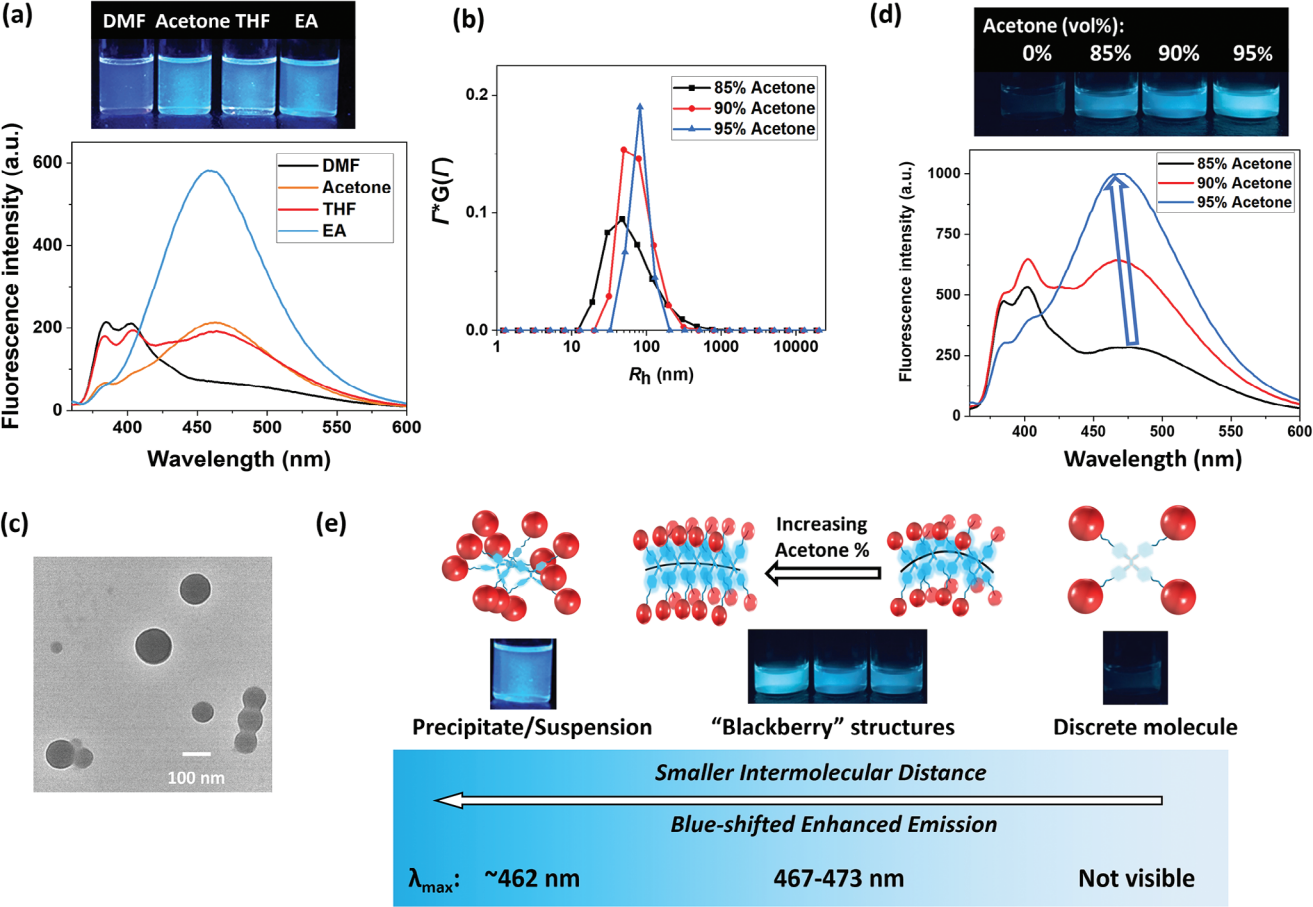
a) (Up) Photograph and (down) fluorescence spectra of 0.03 mg mL^−1^ TPE‐APOSS in different solvents. b) *R*_h_ distributions of 0.3 mg mL^−1^ TPE‐APOSS in DMF‐acetone mixture. c) TEM imaging of the TPE‐APOSS assemblies freeze‐dried from 90 v/v% acetone/DMF solution. d) (Up) Photograph and (down) fluorescence emission spectra of 0.3 mg mL^−1^ TPE‐APOSS in DMF‐acetone mixture. e) The illustration of molecular states and their corresponding fluorescence emission.

To demonstrate the universality of applying ionic AIE macromolecules as sensors for their molecular states, a four‐armed hybrid TPE‐APOSS was also synthesized. Its synthetic route followed that of TPPE‐APOSS and the detailed description as well as characterizations were provided in the supporting information (Scheme S2 and Figures S15–S20, Supporting Information). Due to the lower degree of conjugation of TPE compared to TPPE, TPE‐APOSS absorbs and emits higher energy. When excited at 340 nm (Figure S21, Supporting Information), the DMF solution of TPE‐APOSS was not emissive in the visible range (**Figure** **5**a). Instead, there were two emission peaks at 385 nm and 403 nm, corresponding to the characteristic emission of TPE monomer.^[^ ^54^, ^55^, ^56^ ^]^ The intensity of these two peaks decreased when TPE‐APOSS aggregated (into van der Waals distance) in poor solvents (i.e., acetone, THF, ethyl acetate), accompanied by a new emission peak at ≈462 nm. When acetone was added into its DMF solution, hollow, spherical blackberry structures were also observed (Figure 5c; Figure S22, Supporting Information). Figure 5b demonstrated the increasing size of blackberry structures as the acetone fraction increased from 85% to 95%, similar to the trend from TPPE‐APOSS solutions. These assembly solutions exhibited blue emissions ranging from 473 to 467 nm, again sitting between the single molecule and aggregation states. As summarized in Table 1 and Figure 5e, the emission was also more enhanced and blue‐shifted when TPE‐APOSS was more restricted due to closer intermolecular distances in larger assembly structures. However, because of TPE's lower extent of conjugation and fewer rotational C─C bonds than TPPE, the TPE‐APOSS can only be restricted to limited types of conformations. Therefore, its fluorescence emission was tunable within a narrower window, making the color change more difficult to identify by naked eye compared to TPPE‐APOSS solutions.

### 2.6 Intermolecular Distances and Molecular Conformations in Assembly State Investigated by Molecular Dynamics Simulation

**Figure 6:**
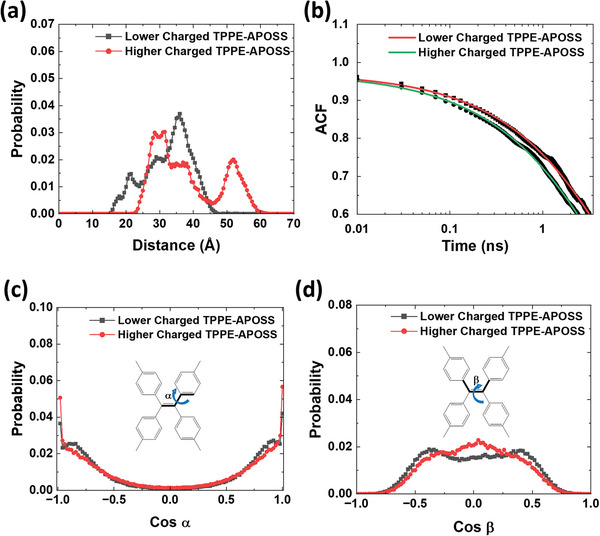
a) Probability distribution of intermolecular distances within self‐assembled TPPE‐APOSS. b) Autocorrelation function of dihedral angle cosα. The symbols and solid lines correspond to ACF function and fitting to Kohlrausch‐Williams‐Watts equation shown in Equation S2 (Supporting Information). c) Probability distribution of dihedral angle cosα, and d) dihedral angle cosβ of self‐assembled TPPE‐APOSS molecules.

To gain a molecular level understanding of the assembly under different conditions as well as their relationship with the fluorescence observations, molecular dynamics simulations were performed on TPPE‐APOSS. This macromolecule was negatively charged through attaching deprotonated carboxylic functional groups to POSS, mirroring the total partial charges of −15 and −13 in 85 v/v% and 95 v/v% acetone/DMF assembly solutions, respectively. Details regarding the simulation method can be found in the S3 (Supporting Information). The intermolecular distances within the blackberry structures were calculated after TPPE‐APOSS molecules self‐assembled, and the ethylenic C═C bond center was considered as reference point for each TPPE‐APOSS molecule. The probability distribution of ethylenic center distances in **Figure** **6**a reveals that the TPPE‐APOSS macromolecules carrying lower charge numbers (in 95 v/v% acetone/DMF) are closer to each other as compared to those of the higher charged ones (in 85 v/v% acetone/DMF): minimum intermolecular distance happens at 16 and 23Å in assembly state, respectively. The closer intermolecular distances in larger blackberry assembly apply more restriction to the TPPE‐APOSS intramolecular mobility, indicated by its longer relaxation time shown in Figure 6b. From the decay correlation function of ethylenic dihedral angle α in TPPE‐APOSS displayed in Figure 6b, the KWW relaxation time τ_KWW_ and the total relaxation time τ are calculated (see **Table** **2**, following Equations (S1)–(S3) in the S3, Supporting Information), revealing that the phenyl ring rotation of the lower charged TPPE‐APOSS takes longer relaxation time in closer mutual distance (larger blackberry structure), leading to the higher fluorescence intensity as observed in Figure 4a.

**Table 2:** KWW relaxation time τkww and total relaxation time τ of dihedral angle α.

	τ_*KWW*_ [*ns*]	τ [*ns*]
Lower charged TPPE‐APOSS assembled in 95% acetone/DMF	10.41	14.26
Higher charged TPPE‐APOSS assembled in 85% acetone/DMF	8.17	11.83

To further understand the reason for blue‐shifted fluorescence emission from larger‐sized TPPE‐APOSS blackberry, the conformation of TPPE‐APOSS in assembly states was extracted from the molecular dynamics simulations, represented by the dihedral angles α and β, highlighted in black in the inset Figure 6c,d. The distribution of dihedral angle cos β is plotted in Figure 6d, where the lower charged TPPE‐APOSS has two cosβ peaks around ± 0.5 (β = 60°), indicating less twisting of its ethylenic C═C bond when restricted in larger blackberry where neighboring molecules are closer, compared to the higher charged counterpart whose cosβ is more distributed around the zero values (β = 90°). From the distribution of dihedral angle cosα as shown in Figure 6c, it is suggested that the phenyl rings of the lower charged TPPE‐APOSS rotate to a larger degree in the assemblies (α>0°), while the cosα of counterpart has higher a probability at ± 1 (α = 0°). Such conformation characteristics of TPPE‐APOSS in larger blackberry led to less centralized electron wavefunction and breakdown of the conjugation,^[^ ^57^ ^]^ both resulting in widened energy gap between HOMO and LUMO, shifting fluorescence emission toward shorter wavelengths.^[^ ^48^, ^57^ ^]^ Therefore, a decrease in the nanoscale intermolecular distance allows for TPPE‐APOSS a lower extent of rotational freedom along with restriction to the molecular conformation that promotes blue‐shifted fluorescence emission, aligning with the experimental observations described earlier.

## 3 Conclusion

The AIE hybrid macromolecule TPPE‐APOSS exhibited strong sensitivity and tunable emission wavelength in response to varying intermolecular distances in solutions, reflecting distinct molecular states. Compared to the yellow, weakly emissive solutions (520 nm) containing individual TPPE‐APOSS, the suspension of TPPE‐APOSS insoluble aggregates exhibited strong, blue‐shifted emission (474–491 nm). Interestingly, the emission intensity and wavelength of the self‐assembled structures into blackberry assemblies, facilitated by electrostatic interaction, fell within the range of 493–508 nm. This was attributed to the partial confinement of the macromolecules in the blackberry structures, which have longer intermolecular distances than in the insoluble aggregates. Especially, the fluorescence emitted by solutions containing larger blackberry structures, indicative of closer intermolecular distances, also occurred at lower wavelengths. The derivate TPE‐APOSS demonstrated a similar capability in sensing the molecular confinement states and intermolecular distances, albeit with lower sensitivity because of its lower degree of conjugation. This study provides a convenient method for detecting changes in intermolecular distances within assemblies in response to external conditions, and the quantitative understanding will pave the way for advancements in designing new fluorophores, supramolecular structures, and nanomaterials, etc.^[^ ^51^, ^58^, ^59^, ^60^, ^61^, ^62^, ^63^, ^64^ ^]^

## Conflict of Interest

The authors declare no conflict of interest.

## Supporting information



Supporting Information

## Data Availability

The data that support the findings of this study are available from the corresponding author upon reasonable request.
