# Supplementary material for: Sensing the Small Change of Intermolecular Distance in Supramolecular Assembly by Using the Tunable Emission Wavelength of AIE‐Active Luminogens
Source: Small. 2024 Oct 24;21(1):2406511. doi: 10.1002/smll.202406511 (PMC11707564; doi:10.1002/smll.202406511)
Supplement: Supplementary file 1 — Supporting Information [file SMLL-21-2406511-s001.pdf]

NANO · MICRO
small

Supporting Information

for *Small*, DOI 10.1002/smll.202406511

Sensing the Small Change of Intermolecular Distance in Supramolecular Assembly by Using the Tunable Emission Wavelength of AIE-Active Luminogens

Xinyu Sun, Hui Li, Abdol Hadi Mokarizadeh, Xiaohan Xu, Tong Liu, Jiancheng Luo, Yuqing Yang, Shuailin Zhang, Fangbei Liu, Mesfin Tsige, Stephen Z. D. Cheng* and Tianbo Liu**

Supporting Information

Sensing the Small Change of Intermolecular Distance in Supramolecular Assembly by Using the Tunable Emission Wavelength of AIE-Active Luminogens

Xinyu Sun,[a] Hui Li, [a] Abdol Hadi Mokarizadeh, [a] Xiaohan Xu, [a] Tong Liu, [a] Jiancheng Luo, [a] Yuqing Yang, [a] Shuailin Zhang, [a] Fangbei Liu, [a] Mesfin Tsige,*[a] Stephen Z. D. Cheng,* [a] Tianbo Liu*[a]

[a] School of Polymer Science and Polymer Engineering, The University of Akron, Akron, Ohio 44325, United States

Table of contents

S1. Sample Characterizations.

S2. Experimental section.

Scheme S1. The synthesis strategy of **TPPE-APOSS**.

Figure S1-S9: ^{1}H NMR and ^{13}C NMR spectra of synthetic compounds for the synthesis of **TPPE-APOSS**.

Figure S10. Normalized UV-vis spectra of TPPE-VPOSS in DMF, THF, and DCM.

Figure S11. IR spectra of TPPE-APOSS in DMF (black) and Acetone (red).

Figure S12. (a) The scattered intensity of TPPE-APOSS in DMF/acetone mixed solvent with various acetone volume fractions. (b) The angular independence and (c) Rg of 0.1mg/mL TPPE-APOSS in 90% Acetone/10% DMF mixed solvent.

Figure S13. TEM imaging of the TPPE-APOSS assemblies freeze-dried from (a) 85 v/v% acetone/DMF and (b) 85 v/v% acetone/DMF, respectively.

Figure S14. (Up) Photograph and (down) fluorescence spectra of 0.04 mg/mL TPPE-VPOSS in DMF-H2O mixed solvents. The maximum emission of TPPE-VPOSS in 20% - 60% H2O solution located at 474 nm.

Scheme S2. The synthesis strategy of **TPE-APOSS**.

Figure S15-S20: ^{1}H NMR and ^{13}C NMR spectra of synthetic compounds for the synthesis of **TPE-APOSS**.

Figure S21. (a) UV absorption and (b) excitation spectra of TPE-APOSS in DMF.

Figure S22. (a) The scattered intensity of 0.3 mg/mL TPE-APOSS in DMF/acetone mixed solvent with various acetone volume fractions. (b) The angular independence and (c) Rg of 0.3mg/mL TPPE-APOSS in 90% Acetone/10% DMF mixed solvent (Rg = 69.8 ± 0.5 nm).

S3. Molecular dynamics simulations.

Table S1. Fitting parameters for Kohlrausch-Williams-Watts equation for dihedral angle α

S1. Sample Characterizations.

The synthesized samples were characterized by NMR, Matrix-assisted laser desorption/ionization time-of flight (MALDI-TOF), and Fourier-transform infrared spectroscopy (FT-IR). ^{1}H and ^{13}C NMR spectra of the samples were obtained in $CDCl_3$ (Sigma-Aldrich, 99.8% D) or DMSO-*d*6 (Sigma-Aldrich, 99.9% D) solvents utilizing Varian Mercury 500 MHz NMR spectrometer under 30 °C. MALDI-TOF mass spectra of the samples were recorded on a Bruker Ultraflex III TOF/TOF mass spectrometer (Bruker Daltonics, Billerica, MA), using salt and DCTB matrix. FT-IR spectra were obtained from Perkin Elmer Fourier Transform Infrared Spectroscopy equipped with ATR accessory.

The self-assembly of the TPPE-APOSS and TPE-APOSS macromolecules were monitored by Laser Light Scattering. A commercial Brookhaven Instruments LLS spectrometer equipped with a solid-state laser operating at 532 nm was used for both Static Light Scattering (SLS) and Dynamic Light Scattering (DLS) measurement. DLS measures the intensity−intensity time correlation function by means of a BI-9000AT multichannel digital correlator. The field correlation function $|g^{(1)}(\tau)|$ was analyzed by the constrained regularized CONTIN method to yield information on the distribution of the characteristic line width Γ. The normalized distribution function of the characteristic line width, $G(\Gamma)$ can be used to determine an average apparent translational diffusion coefficient, $D_{app} = \Gamma/q^2$, where q is the scattering factor. The hydrodynamic radius R_h is related to D via the Stokes−Einstein equation: $R_h = kT/(6\pi\eta D)$ where k is the Boltzmann constant and η the viscosity of the solvent at temperature T. From DLS measurements, the particle-size distribution in solution is obtained from a plot of $\Gamma G(\Gamma)$ vs R_h. SLS experiments were performed at scattering angles (θ) between 40° and 90°, at 2° intervals. Derived from Rayleigh−Gans−Debye equation, a Zimm plot was used to analyze the SLS data to obtain the radius of gyration (R_g) of the assemblies.

The UV-vis spectra were taken by Agilent Cary 60 UV-Vis Spectrometer. The fluorescence emission spectra were collected by Agilent Cary Eclipse Fluorescence Spectrometer. Transmission Electron Microscope (TEM) images were collected using a JEOL-1230 electron microscope with an accelerating voltage of 120 kV. A droplet of 8 µL sample solution was placed onto a carbon film coated copper grid (Ted Pella, Inc.) and freeze-dried for 2 days prior to taking TEM images. Atomic Force Microscopy (AFM) experiments were conducted on a Dimension Icon Atomic Force Microscopy (Bruker). Samples were prepared by drop-casting 10 µL onto the acetone rinsed Si wafer substrates. After overnight solvent evaporation under vacuum and chilled conditions, images were scanned in the tapping mode and analyzed by NanoScope Analysis software.

S2. Experimental section

Materials.

Chemicals and solvents were used as received from Sigma-Aldrich and Tokyo Chemical Industry Co., Ltd.. Monofunctionalized polyhedral oligomeric silsesquioxane cages such as VPOSS-OH and VPOSS-S-OH were synthesized as reported.[1,2]

Synthetic procedures.

Scheme S1. The synthesis strategy of **TPPE-APOSS**.

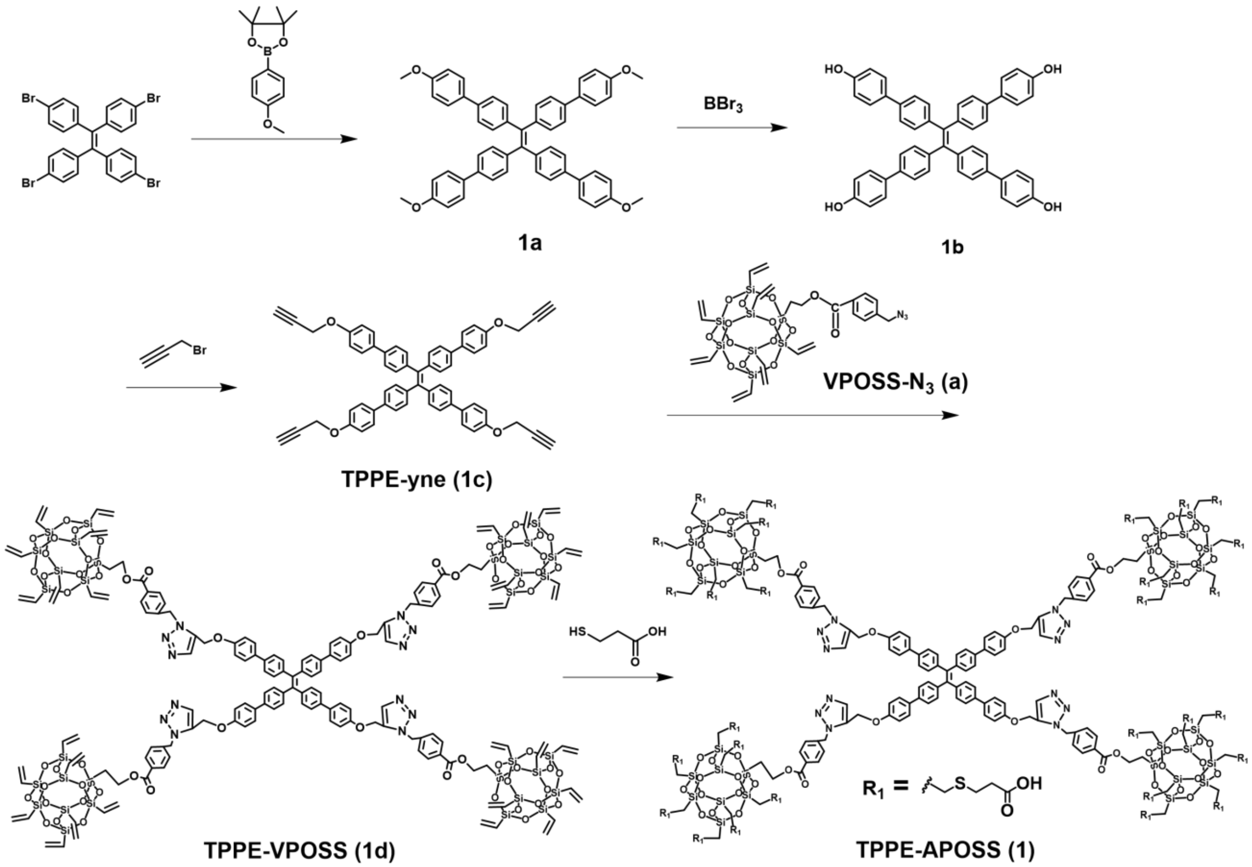

Synthesis of 4’,4’’’,4’’’ ”,4’’’ ’’’ ”-(Ethene-1,1,2,2-tetrayl) tetrakis (([1,1’-biphenyl]-4-methoxy)) (1a):

Under nitrogen atmosphere, 1,1,2,2-Tetrakis (4-bromophenyl) ethylene (3 mmol), 4-methoxyphenylboronic acid pinacol ester (5 mmol), and tetrakis (triphenylphosphine) palladium (0.1 mmol) were added to the vessel. To the vessel via syringe were added 100 mL of degassed *N,N*-dimethylformamide (DMF) and then 5 mmol of potassium carbonate dissolved in 5 mL of de-ionized water under room temperature. Then the reaction mixture was allowed to heat at 100 °C and refluxed under nitrogen for 60 h. After the reaction, the vessel was brought to RT, and the contents were collected by filtration. The filtrate was subjected to wash with water and extracted by dichloromethane for three times, followed by drying over $MgSO_4$ and concentration *via* rotary evaporation. The crude material was subjected to chromatography on silica gel (dichloromethane/hexane= 4:1) to afford yellow product in 90% yield. ^{1}H NMR (500 MHz, Chloroform-*d*) δ 7.51 (d, *J* = 8.8 Hz, 8H), 7.35 (d, *J* = 8.4 Hz, 8H), 7.15 (d, *J* = 8.3 Hz, 8H), 6.93 (d, *J* = 8.8 Hz, 8H), 3.83 (s, 12H).

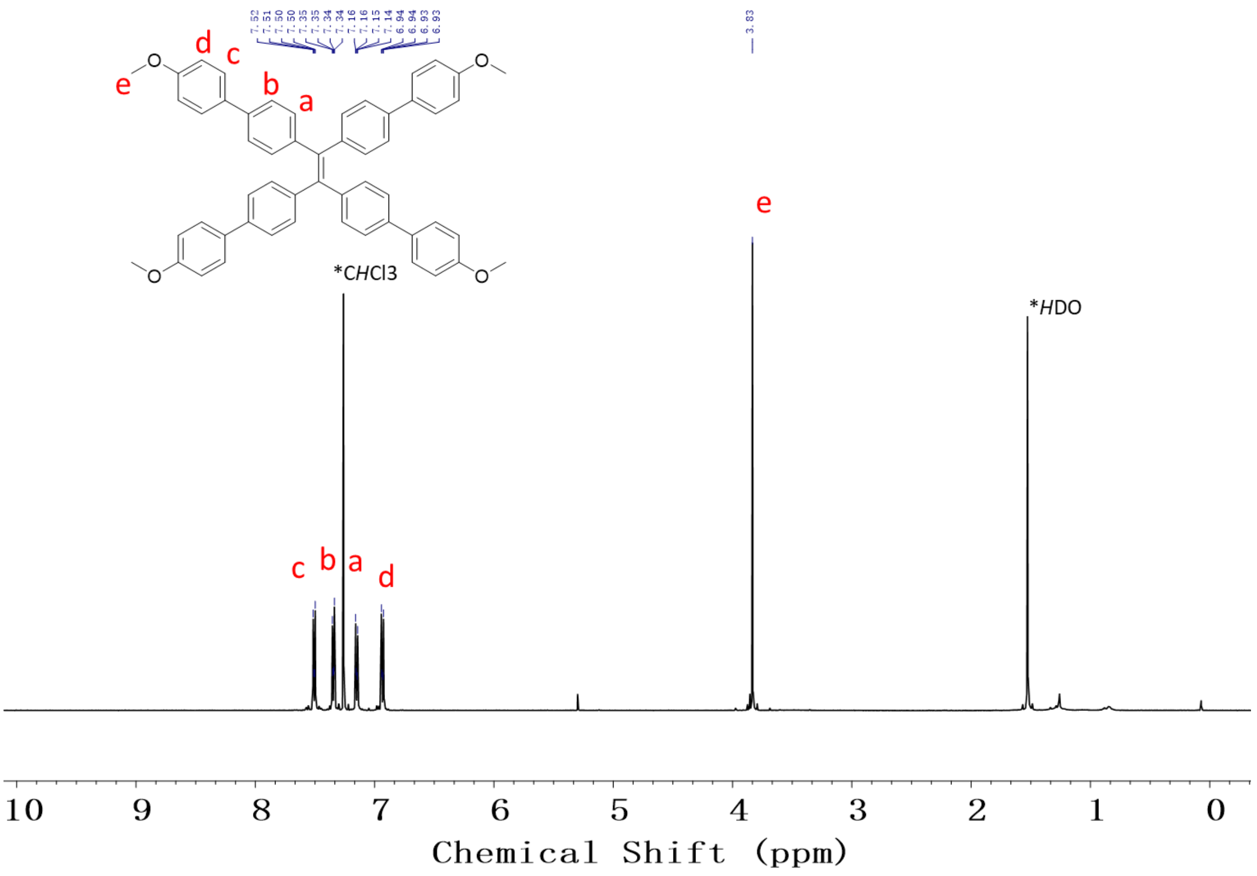

Figure S1. ^{1}H NMR spectrum of compound **1a** in $CDCl_3$.

Synthesis of 4’,4’’’,4’’’ ’’,4’’’ ’’’ ’’-(Ethene-1,1,2,2-tetrayl) tetrakis (([1,1’-biphenyl]-4-hydroxy)) (1b):

10 mmol of BBr_3 was added dropwise to a cooled solution of **1a** (1.4 mmol) in 1 L of anhydrous dichloromethane. After removal of the cooling bath, the resulting deep green then dark solution was stirred at room temperature for 18 h. After the reaction, the mixture was hydrolyzed under ice-cooling by dropwise addition of water. The precipitate was collected by filtration, washed with water, and extracted by dichloromethane for three times. Then the desired product was obtained through chromatography on silica gel (dichloromethane/methanol= 10:1) to afford yellow solid in 80% yield. ^{1}H NMR (500 MHz, DMSO-d_6) δ 9.50 (s, 4H), 7.45 (d, J = 8.7 Hz, 8H), 7.41 (d, J = 8.4 Hz, 8H), 7.07 (d, J = 8.4 Hz, 8H), 6.79 (d, J = 8.7 Hz, 8H).

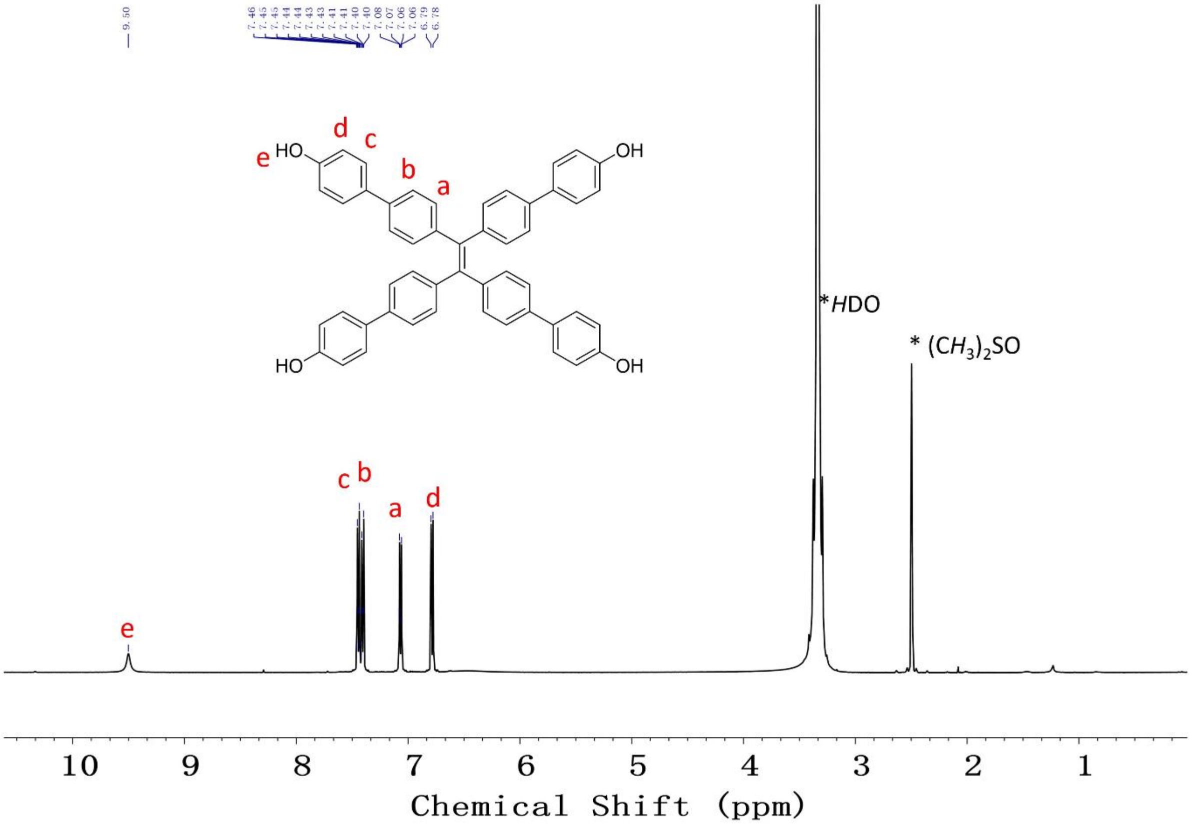

Figure S2. ^{1}H NMR spectrum of compound **1b** in DMSO-d_6.

Synthesis of 4’,4’’’,4’’’ ”,4’’’ ’’’ ”-(Ethene-1,1,2,2-tetrayl) tetrakis (([1,1’-biphenyl]-4-methoxy-1-propyne)) (1c):

The mixture of compound **1b** (1 mmol), propargyl bromide (10 mmol), and potassium carbonate (5 mmol) in anhydrous DMF was vigorously stirred under nitrogen atmosphere at 70 °C for 24 h. The mixture was then cooled to room temperature and the contents were collected by filtration. The filtrate was dissolved in DCM and washed with water for three times. After removal of the solvent *via* rotary evaporation, the crude product was subjected to chromatography on silica gel (chloroform/hexane 10:2) to afford yellow solid in 62% yield.^{1}H NMR (500 MHz, Chloroform-*d*) δ 7.52 (d, *J* = 8.7 Hz, 8H), 7.34 (d, *J* = 8.1 Hz, 8H), 7.15 (d, *J* = 8.3 Hz, 8H), 7.01 (d, *J* = 8.7 Hz, 8H), 4.72 (d, *J* = 2.4 Hz, 8H), 2.52 (t, *J* = 2.4 Hz, 4H). ^{13}C NMR (500MHz, DMSO-d_6) δ 156.82, 141.94, 139.84, 137.57, 132.45, 131.43, 127.49, 125.53, 115.26, 79.23, 78.23, 55.47.

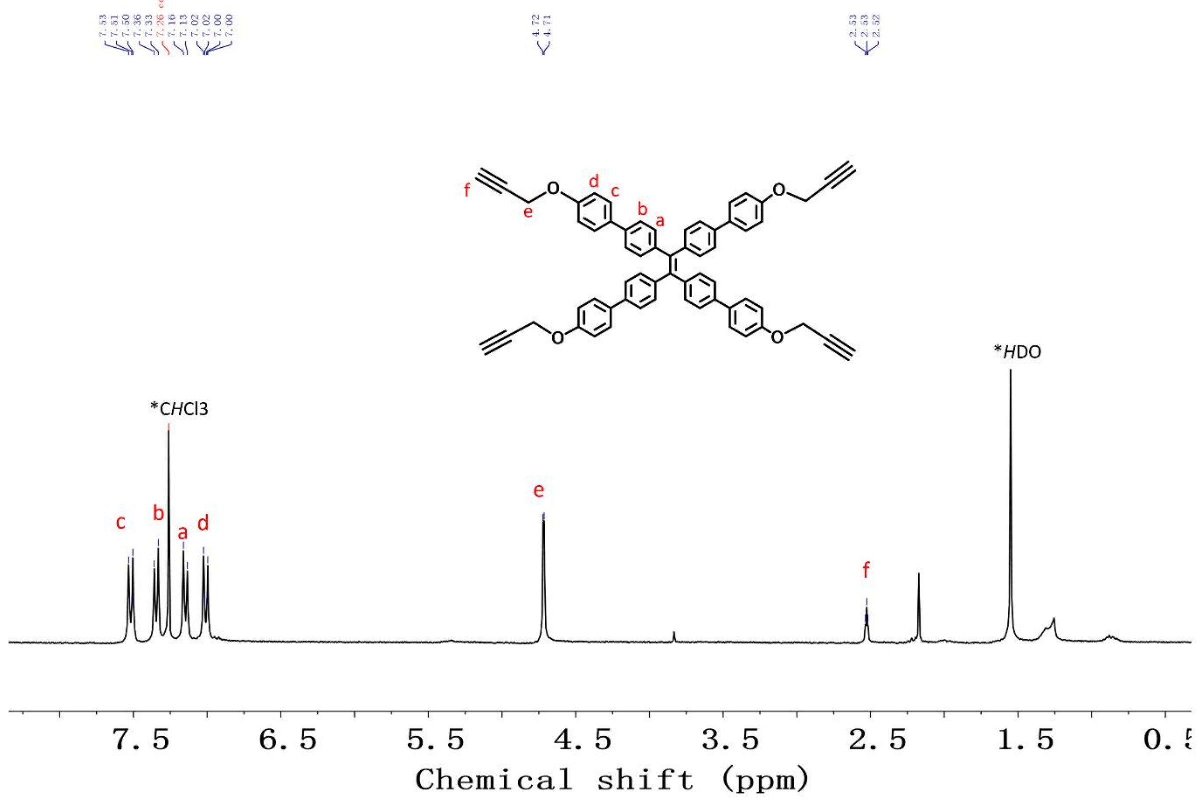

Figure S3. ^{1}H NMR spectrum of compound **1c** in $CDCl_3$.

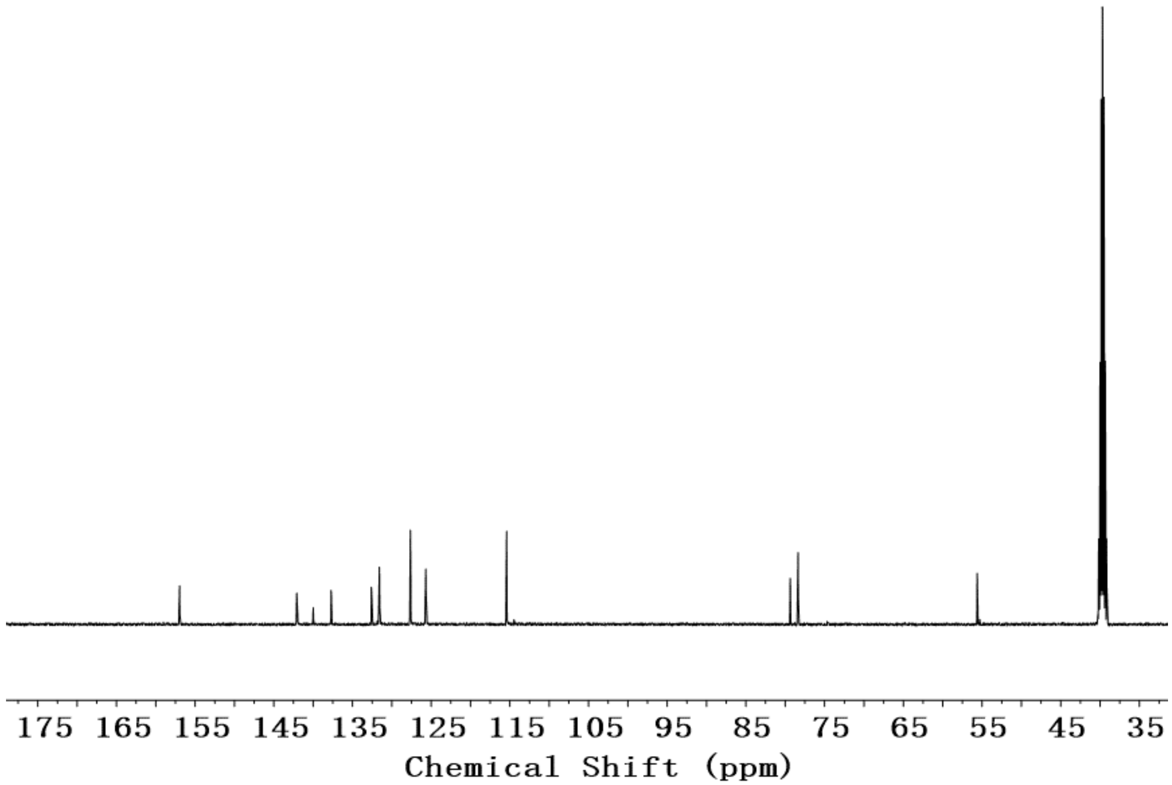

Figure S4. ^{13}C NMR spectrum of compound **1c** in $CDCl_3$.

Synthesis of VPOSS-N_3 (a):

In a 20 mL vial with a magnetic stirring bar, VPOSS-OH (1 mmol), 4-(Azidomethyl) benzoic acid (1.5 mmol), and DMAP (0.5 mmol) were fully dissolved in 10 mL DCM. Then the solution was cooled to 0 ℃, followed by the dropwise addition of DIC (3 mmol) via syringe. The mixture was allowed to warm up to room temperature and stirred for another 24 h. After the reaction, the precipitate was filtered the solvent was removed *via* rotary evaporation. The crude product was subjected to chromatography on silica gel (chloroform/Ethyl Acetate 10:1) as eluent to afford white solid in 75% yield. 1H NMR (500 MHz, Chloroform-d) δ 8.06 (d, 2H), 7.36 (d, 2H), 6.17 – 5.82 (m, 21H), 4.52 – 4.45 (m, 2H), 4.40 (s, 2H), 1.40 – 1.31 (m, 2H).

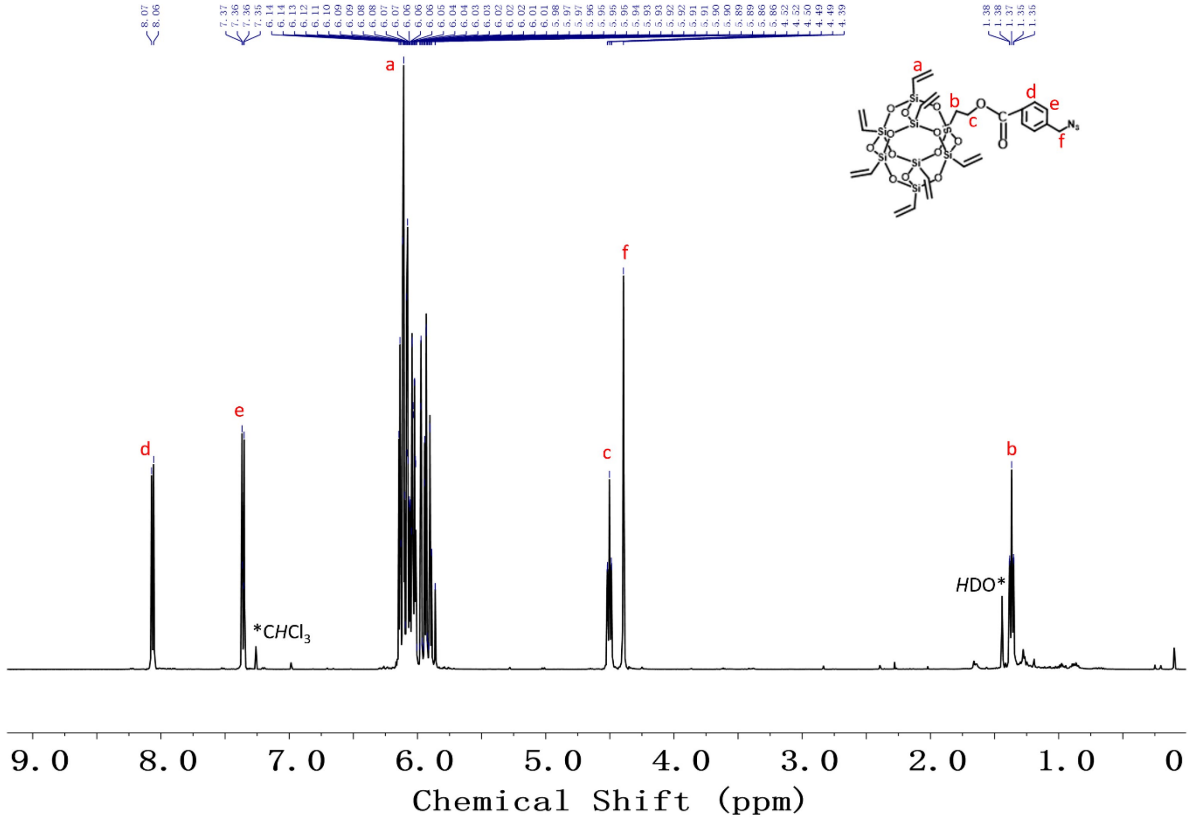

Figure S5. ^{1}H NMR spectrum of compound **VPOSS-N_3 (a)** in $CDCl_3$.

Synthesis of TPPE-VPOSS (1d):

In a 100 mL Schlenk flask, 0.07 mmol of VPOSS-N_3 **(a)** and 0.016 mmol of **1c** were fully dissolved in 10 mL of freshly distilled THF. The resulting solution was degassed by freeze-pump-thaw cycles for three times. After that, 0.02 mmol of CuBr and 0.07 mmol of PMDETA were added via pipet under nitrogen atmosphere. The mixture was further degassed by another one cycle of freeze-pump-thaw and then stirred at room temperature for 12 h. After the reaction, the mixture was filtered and evaporated *via* rotary evaporation before purification through flash column chromatography on the silica gel (dichloromethane: methanol 10:1) to afford yellow product in 92% yield. ^{1}H NMR (500 MHz, Chloroform-*d*) δ 8.04 (d, *J* = 8.3 Hz, 8H), 7.54 (s, 4H), 7.49 (d, *J* = 8.8 Hz, 8H), 7.31 (dd, *J* = 17.2, 8.3 Hz, 16H), 7.14 (d, *J* = 8.3 Hz, 8H), 6.99 (d, 9H), 6.15 – 5.82 (m, 84H), 5.57 (s, 8H), 5.22 (s, 8H), 4.48 (t, *J* = 8.6, 7.5 Hz, 8H), 1.34 (t, *J* = 8.6, 7.4 Hz, 8H). ^{13}C NMR (500 MHz, Chloroform-*d*) δ 165.89, 157.84, 145.05, 142.57, 140.42, 139.24, 138.53, 137.22, 133.98, 132.07, 131.11, 130.58, 128.73, 128.64, 128.10, 125.99, 122.76, 115.15, 62.32, 61.67, 53.96, 13.23. MS (MALDI-TOF): calcd. average mass for $[M]^{+}$: 4093.3 Da, found 4093.4 Da.

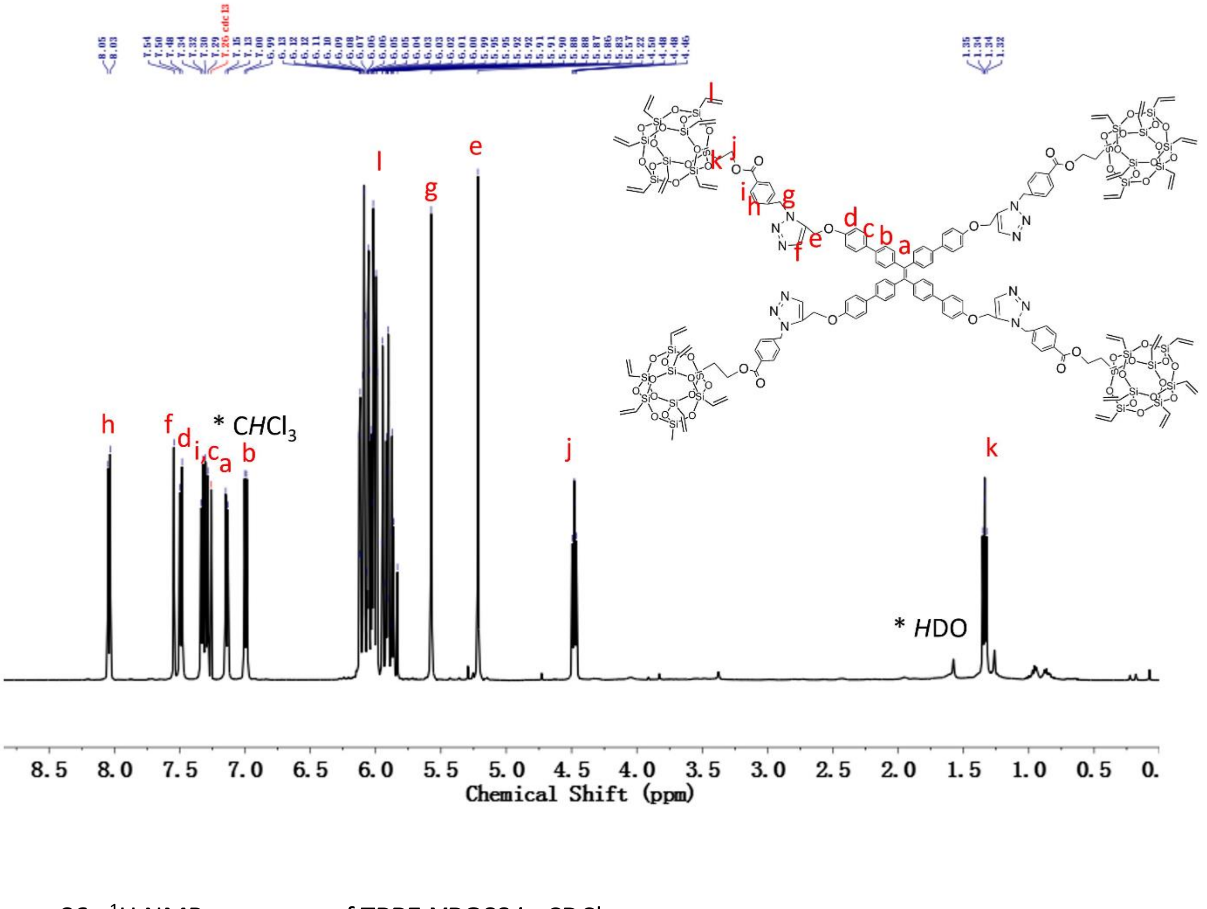

Figure S6. ^{1}H NMR spectrum of **TPPE-VPOSS** in $CDCl_3$.

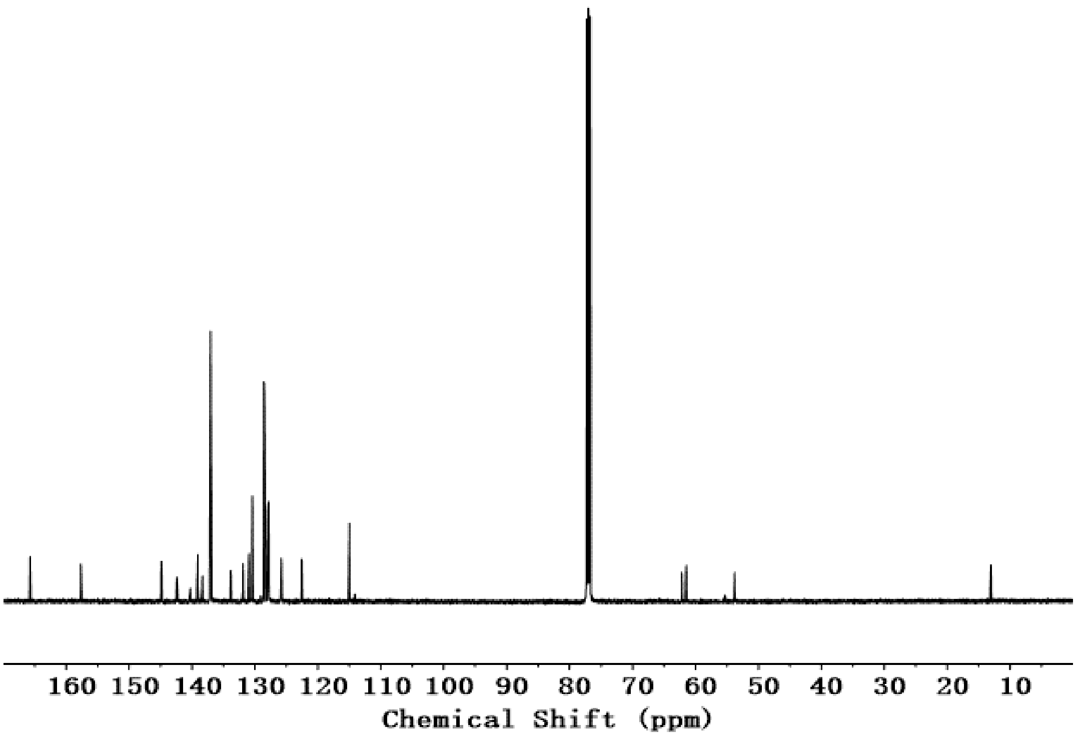

Figure S7. ^{13}C NMR spectrum of **TPPE-VPOSS** in $CDCl_3$.

Synthesis of TPPE-APOSS (1):

0.007 mmol of **TPPE-VPOSS (1d)**, 0.5 mmol of 3-Mercaptopropionic acid, and the photoinitiator Irgacure 2959 (0.02 mmol) were added into an open vial with 4 mL of THF. The reaction was conducted under UV irradiation (365 nm) for 30 min. Then the solvent was dried and replaced by 2 mL of DMSO. Another 0.5 mmol of 3-Mercaptopropionic acid and 0.02 mmol of Irgacure 2959 were added to ensure the complete reaction. The mixture was then washed and precipitated by diethyl ether for multiple times to yield yellow product in 95% yield. ^{1}H NMR (500 MHz, DMSO-d_6) δ 8.30 (s, 4H), 7.95 (d, *J* = 7.7 Hz, 8H), 7.57 (d, *J* = 8.3 Hz, 8H), 7.44 (dd, *J* = 28.0, 8.4 Hz, 16H), 7.08 (dd, *J* = 24.6, 8.2 Hz, 16H), 5.71 (s, 8H), 5.14 (s, 8H), 4.41 (s, 8H), 3.04 – 2.30 (m, 168H), 1.46 – 0.74 (m, 64H). ^{13}C NMR (126 MHz, DMSO-*d*6) δ 172.92, 165.12, 157.71, 143.02, 141.91, 141.14, 137.59, 132.05, 131.43, 129.55, 128.03, 127.51, 125.46, 124.90, 115.08, 61.17, 55.14, 52.41, 46.74, 45.21, 34.32, 30.72, 26.16, 25.03, 12.33, 3.64.

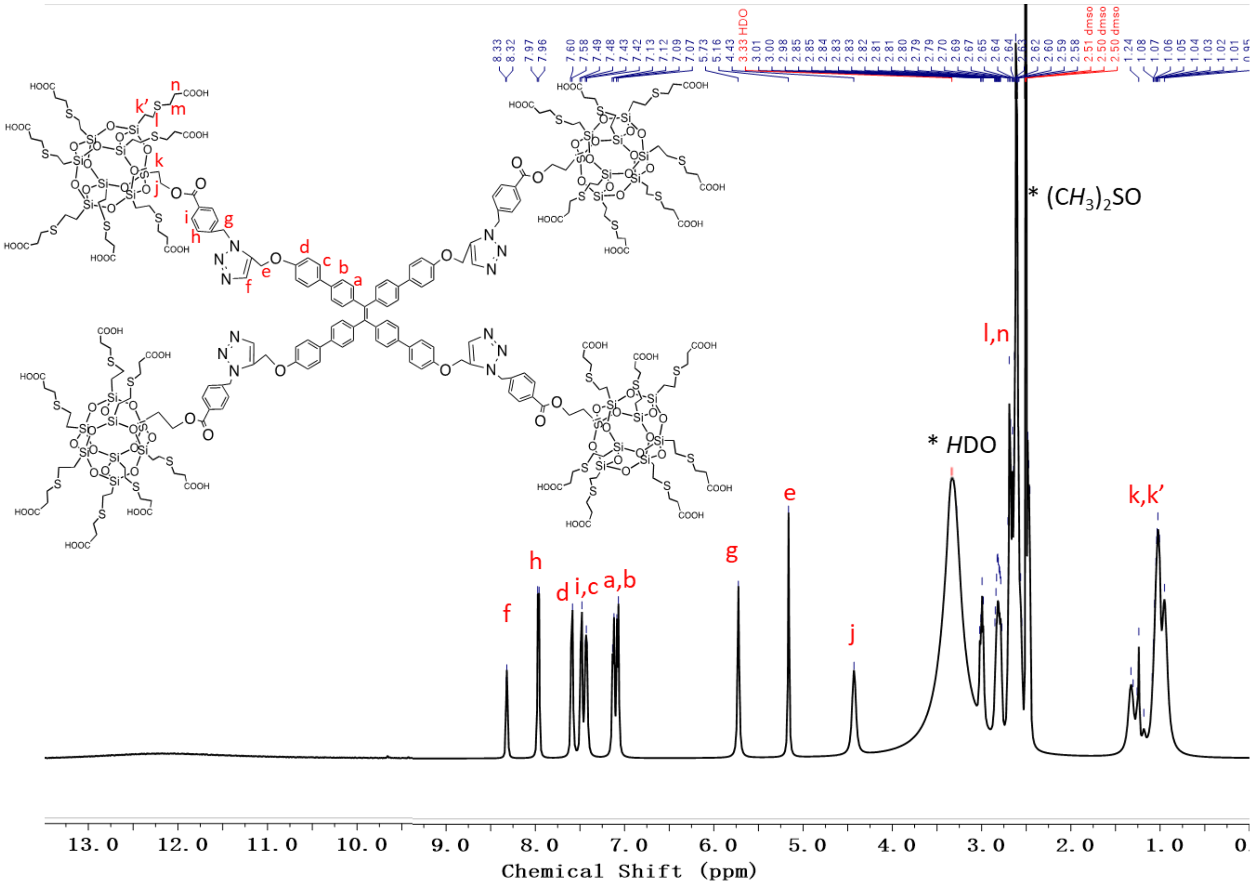

Figure S8. ^{1}H NMR spectrum of **TPPE-APOSS** in DMSO-d_6.

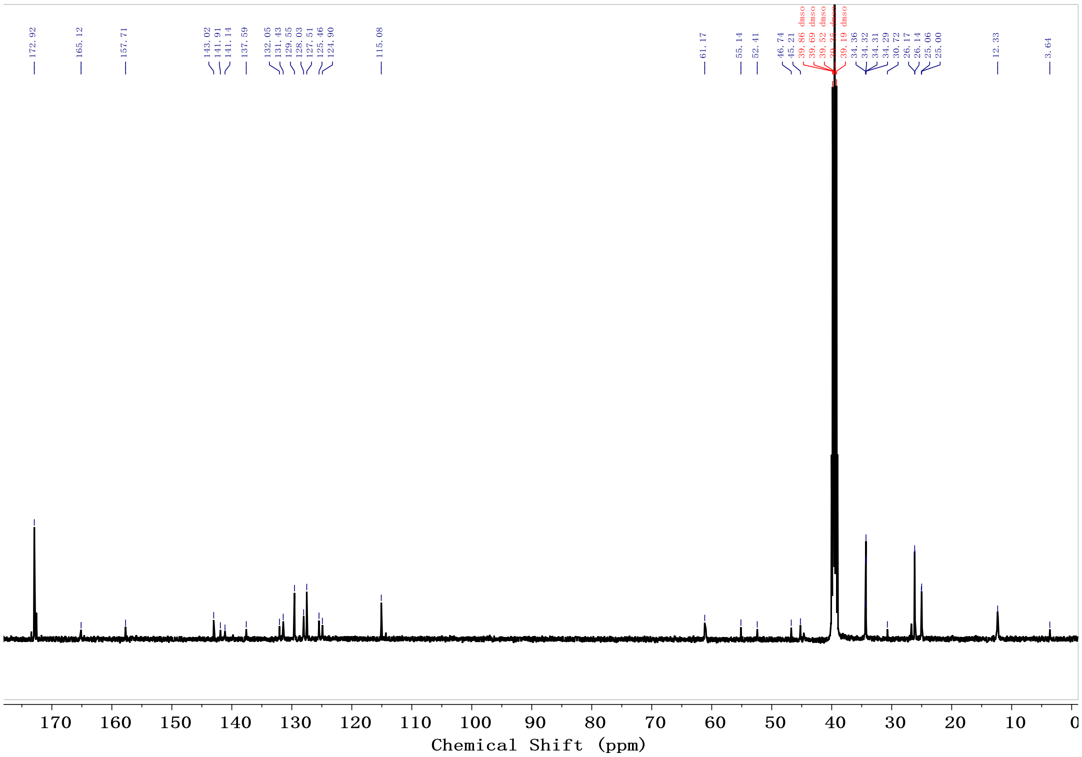

Figure S9. ^{13}C NMR spectrum of **TPPE-APOSS** in DMSO-d_6.

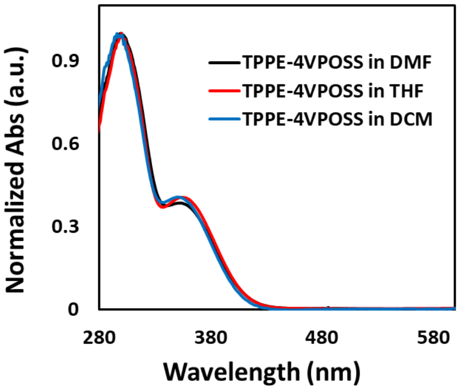

Figure S10. Normalized UV-vis spectra of TPPE-VPOSS in DMF, THF, and DCM.

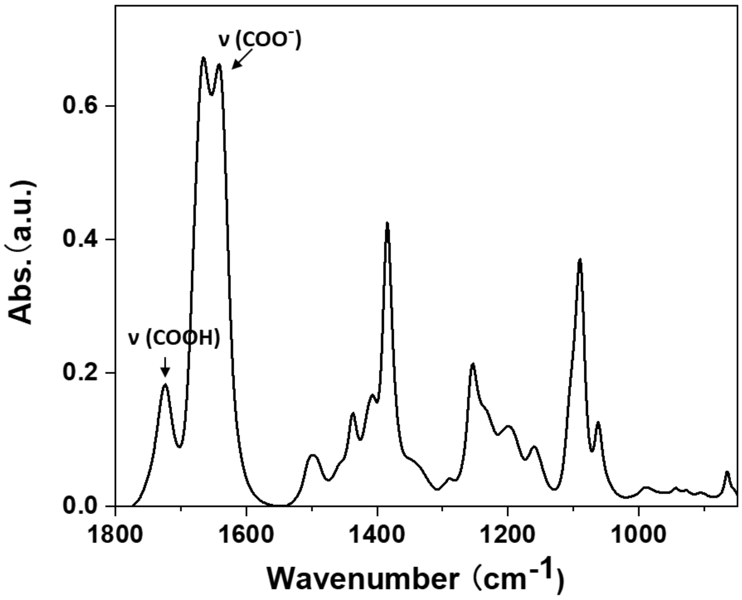

Figure S11. In situ FT-IR spectra of APOSS in DMF.

In the DMF solution of APOSS, the C=O stretching of -COOH groups locates at ν ≈ 1724 cm^{-1}, and the asymmetric stretching band of the ionized carboxylate ($-COO^-$) groups located at 1641 cm^{-1}. The band between them at ~1666 cm^{-1} belongs to the CO stretching of the DMF solvent. To tell the relative intensity of -COOH and $-COO^-$ groups, gaussian fitting of each peak was applied. Therefore, the degree of deprotonation (α) can be estimated from the ratio of the intensity of the $\nu(COO^-)$ peak to the sum of the intensities of the $\nu(COO^-)$ and ν(COOH) peaks[3]: $\alpha = \frac{I_{\nu(COO^-)}}{I_{\nu(COO^-)} + I_{\nu(COOH)}}$.

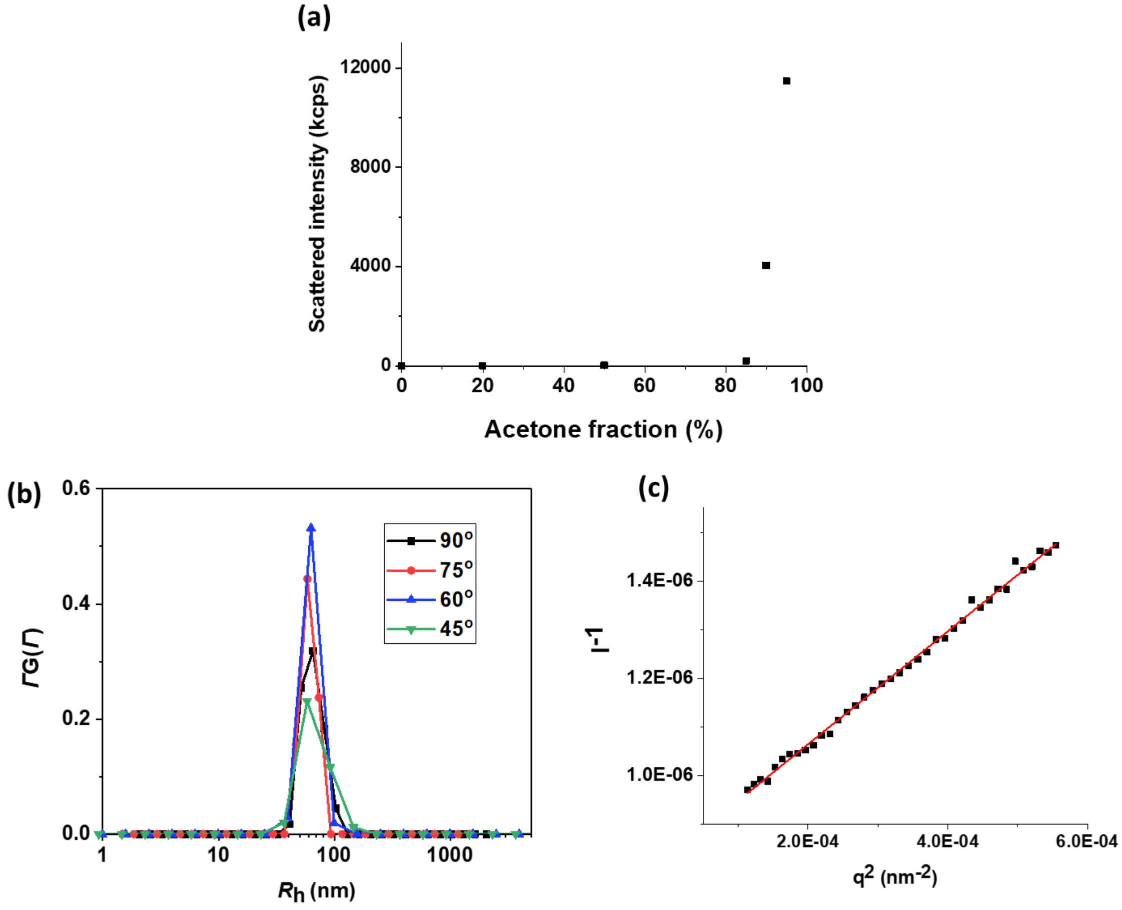

Figure S12. (a) The scattered intensity of TPPE-APOSS in DMF/acetone mixed solvent with various acetone volume fractions. (b) The angular independence and (c) R_g of 0.1mg/mL TPPE-APOSS in 90% Acetone/10% DMF mixed solvent.

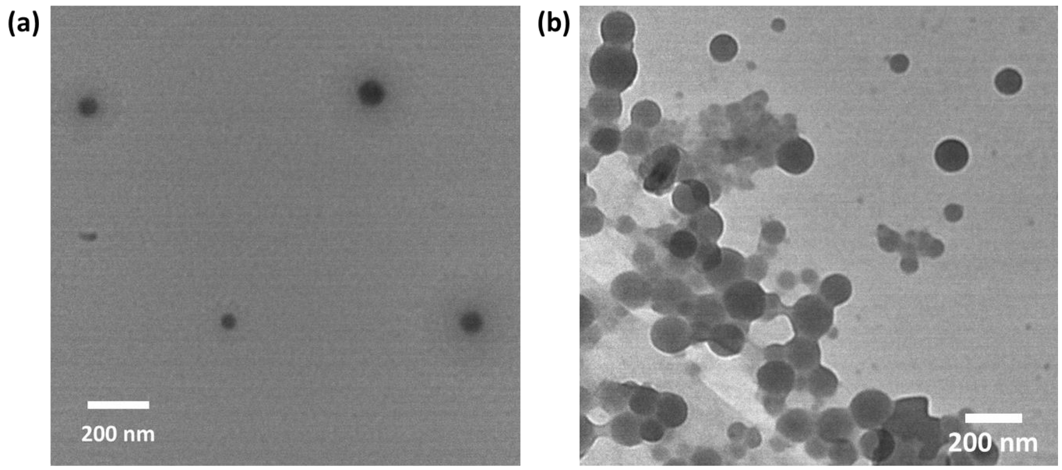

Figure S13. TEM imaging of the TPPE-APOSS assemblies freeze-dried from (a) 85 v/v% acetone/DMF and (b) 85 v/v% acetone/DMF, respectively.

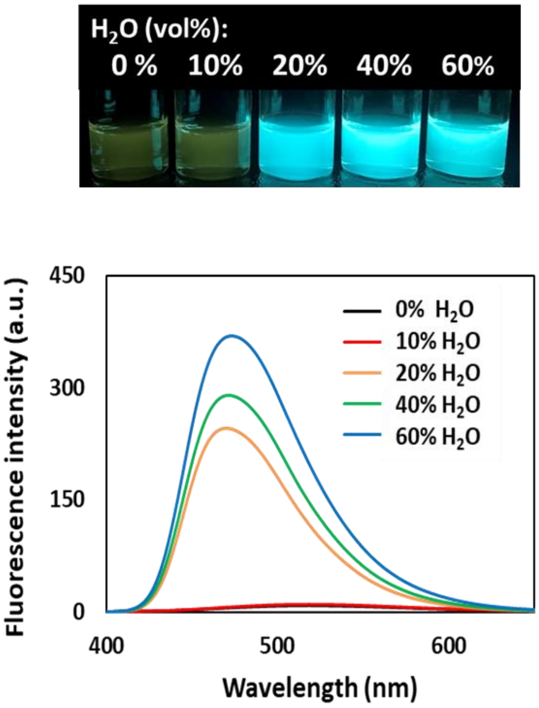

Figure S14. (Up) Photograph and (down) fluorescence spectra of 0.04 mg/mL TPPE-VPOSS in DMF-H_2O mixed solvents. The maximum emission of TPPE-VPOSS in 20% - 60% H_2O solution located at 474 nm.

Scheme S2. The synthesis strategy of **TPE-APOSS**.

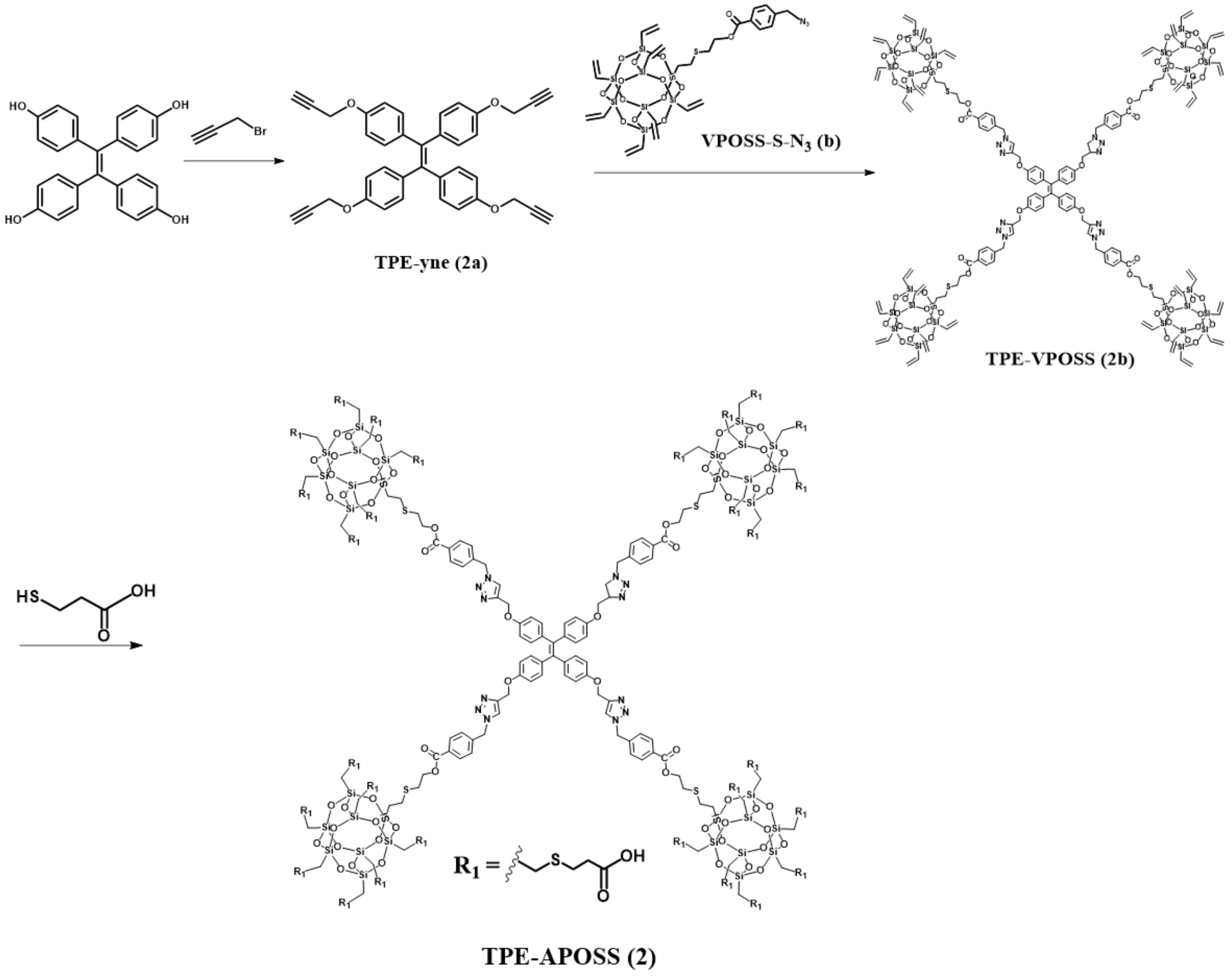

Synthesis of compound 2a:

The mixture of 4,4′,4′′,4′′′-(Ethene-1,1,2,2-tetrayl) tetraphenol (2 mmol), propargyl bromide (20 mmol), and potassium carbonate (10 mmol) in anhydrous DMF was vigorously stirred under nitrogen atmosphere at 70 °C for 24 h. The mixture was then cooled to room temperature and the contents were collected by filtration. The filtrate was dissolved in DCM and wash with water for three times. After removal of the solvent *via* rotary evaporation, the crude product was subjected to chromatography on silica gel (chloroform/hexane 10:2) to yield white solid in 85% yield. ^{1}H NMR (500 MHz, Chloroform-*d*) δ 6.93 (d, *J* = 8.7 Hz, 4H), 6.70 (d, *J* = 8.8 Hz, 4H), 4.62 (d, *J* = 2.4 Hz, 8H), 2.50 (t, *J* = 2.4 Hz, 4H).

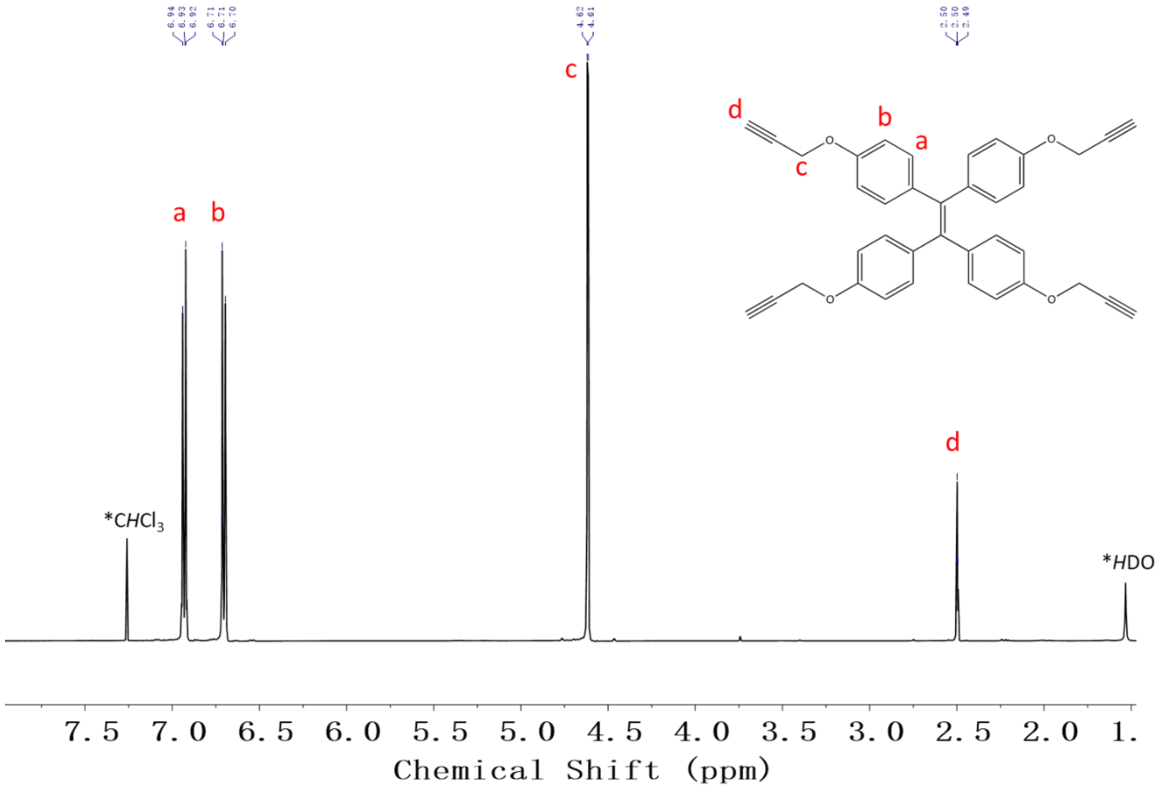

Figure S15. ^{1}H NMR spectrum of compound **2a** in $CDCl_3$.

Synthesis of compound VPOSS-S-N_3 (b):

In a 20 mL vial with a magnetic stirring bar, VPOSS-S-OH (1 mmol), 4-(Azidomethyl) benzoic acid (1.5 mmol), and DMAP (0.5 mmol) were fully dissolved in 10 mL DCM. Then the solution was cooled to 0 ℃, followed by the dropwise addition of DIC (3 mmol) via syringe. The mixture was allowed to warm up to room temperature and stirred for another 24 h. After the reaction, the precipitate was filtered the solvent was removed *via* rotary evaporation. The crude product was subjected to chromatography on silica gel (chloroform/Ethyl Acetate 10:1) as eluent to afford white solid in 75% yield. ^{1}H NMR (500 MHz, Chloroform-*d*) δ 8.06 (d, *J* = 8.3 Hz, 1H), 7.39 (d, *J* = 8.2 Hz, 1H), 6.26 – 5.78 (m, 15H), 4.52 – 4.35 (m, 3H), 2.89 (t, *J* = 7.0 Hz, 2H), 2.77 – 2.69 (m, 2H), 1.18 – 1.03 (m, 2H).

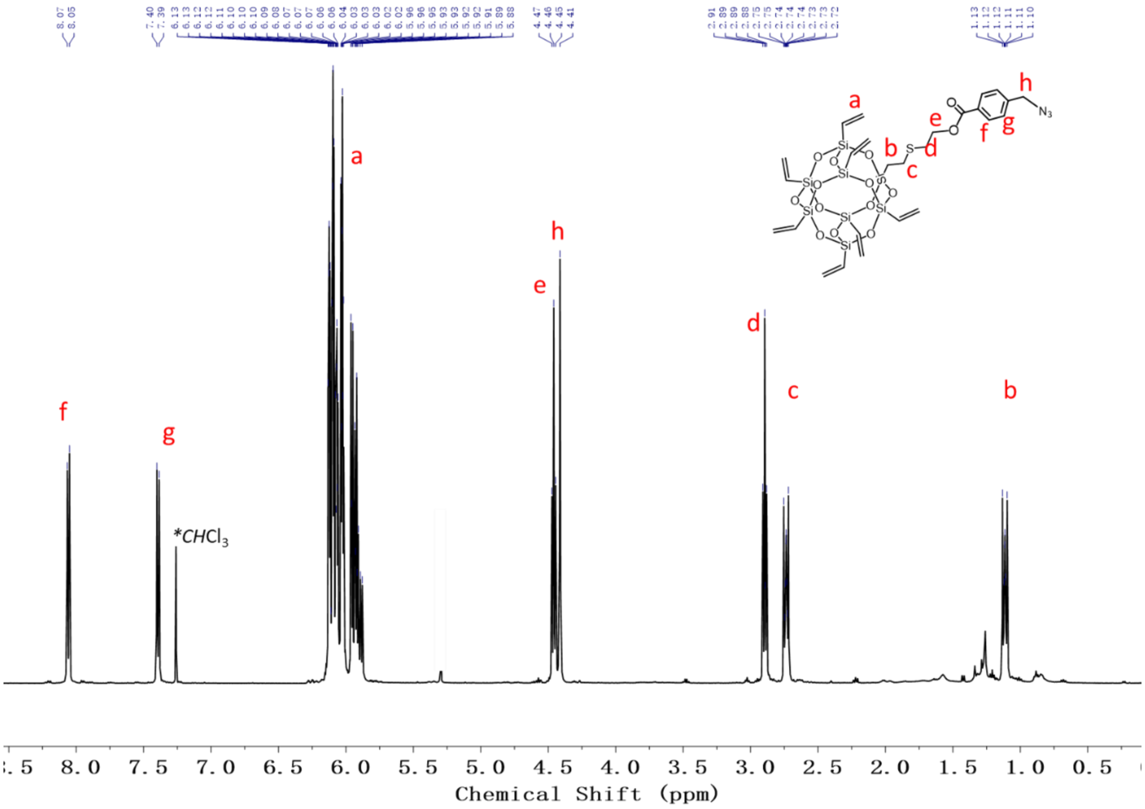

Figure S16. ^{1}H NMR spectrum of compound **VPOSS-S-N_3 (b)** in $CDCl_3$.

Synthesis of compound TPE-VPOSS (2b):

In a 100 mL Schlenk flask, 0.05 mmol of **b** and 0.01 mmol of **2a** were fully dissolved in 10 mL of freshly distilled THF. The resulting solution was degassed by freeze-pump-thaw cycles for three times. After that, 0.02 mmol of CuBr and 0.07 mmol of PMDETA were added via pipet under nitrogen atmosphere. The mixture was further degassed by another one cycle of freeze-pump-thaw and then stirred at room

temperature for 12 h. After the reaction, the mixture was filtered and evaporated *via* rotary evaporation before purification through flash column chromatography on the silica gel (dichloromethane: methanol 10:1) to afford light yellow product in 79% yield. ^{1}H NMR (500 MHz, Chloroform-*d*) δ 8.04 (d, *J* = 8.4 Hz, 8H), 7.55 (s, 4H), 7.31 (d, *J* = 8.0 Hz, 8H), 6.90 (d, *J* = 8.3 Hz, 8H), 6.68 (d, *J* = 8.4 Hz, 8H), 6.17 – 5.84 (m, 84H), 5.58 (s, 8H), 5.11 (s, 8H), 4.44 (t, *J* = 7.0 Hz, 8H), 2.88 (t, *J* = 7.1 Hz, 8H), 2.72 (t, *J* = 8.6 Hz, 8H), 1.10 (t, 8H). ^{13}C NMR (500 MHz, Chloroform-*d*) δ 165.74, 156.74, 145.05, 139.59, 138.77, 137.20, 132.70, 130.71, 130.60, 128.79, 128.75, 128.04, 122.89, 114.08, 64.07, 62.09, 53.92, 30.44, 26.50, 13.39. MS (MALDI-TOF): calcd. average mass for $[M]^+$: 4031.2 Da, found: 4031.0 Da.

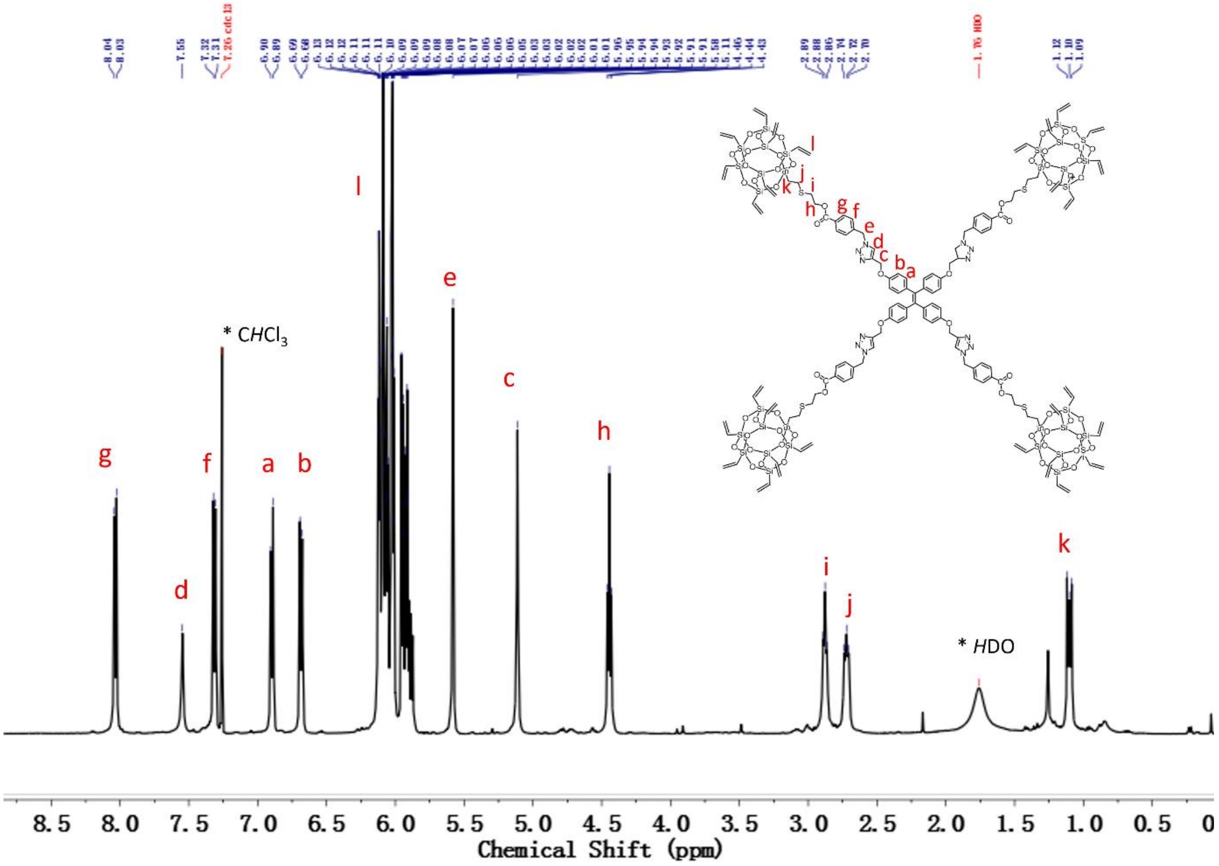

Figure S17. ^{1}H NMR spectrum of **TPE-VPOSS** in $CDCl_3$.

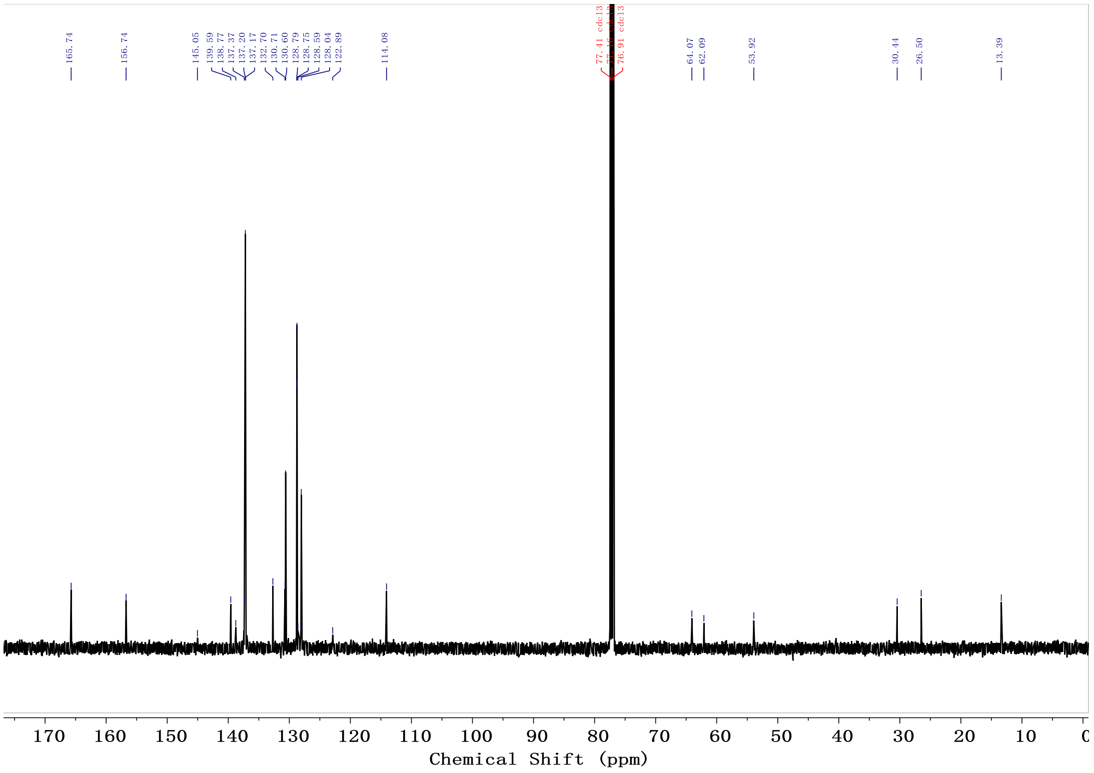

Figure S18. ^{13}C NMR spectrum of **TPE-VPOSS** in $CDCl_3$.

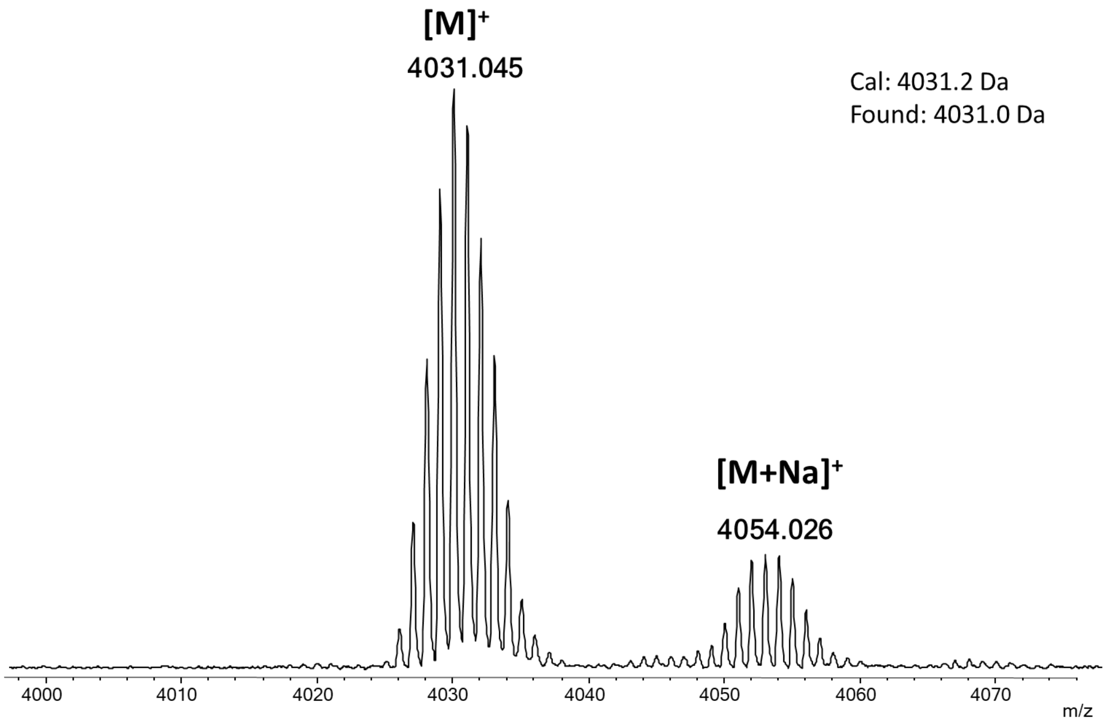

Figure S19. MALDI-TOF mass spectroscopy characterization of **TPE-VPOSS**: calcd: 4031.2 Da; found: 4031.0 Da.

Synthesis of compound 2c:

0.007 mmol of **TPE-VPOSS**, 0.5 mmol of 3-Mercaptopropionic acid, and the photoinitiator Irgacure 2959 (0.02 mmol) were added into an open vial with 4 mL of THF. The reaction was conducted under UV irradiation (365 nm) for 30 min. Then the solvent was dried and replaced by 2 mL of DMSO. Another 0.5 mmol of 3-Mercaptopropionic acid and 0.02 mmol of Irgacure 2959 were added to ensure the complete reaction. The mixture was then washed and precipitated by diethyl ether for multiple times to yield yellow product in 95% yield. ^{1}H NMR (500 MHz, DMSO-d_6) δ 8.30 (s, 4H), 7.95 (d, *J* = 7.7 Hz, 8H), 7.57 (d, *J* = 8.3 Hz, 8H), 7.44 (dd, *J* = 28.0, 8.4 Hz, 16H), 7.08 (dd, *J* = 24.6, 8.2 Hz, 16H), 5.71 (s, 8H), 5.14 (s, 8H), 4.41 (s, 8H), 3.04 – 2.30 (m, 168H), 1.46 – 0.74 (m, 64H). ^{13}C NMR (126 MHz, DMSO-*d*6) δ 172.92, 165.12, 157.71, 143.02, 141.91, 141.14, 137.59, 132.05, 131.43, 129.55, 128.03, 127.51, 125.46, 124.90, 115.08, 61.17, 55.14, 52.41, 46.74, 45.21, 34.32, 30.72, 26.16, 25.03, 12.33, 3.64.

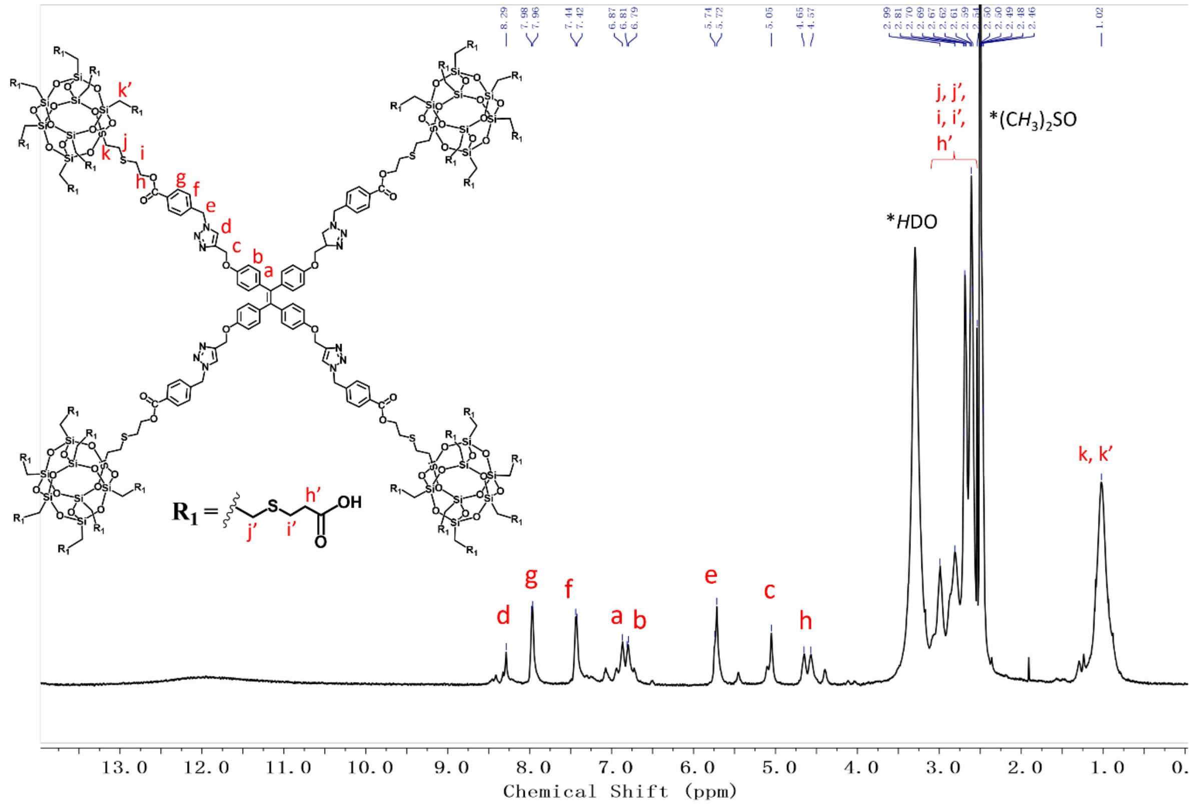

Figure S20. ^{1}H NMR spectrum of compound **TPE-APOSS** in DMSO-d_6.

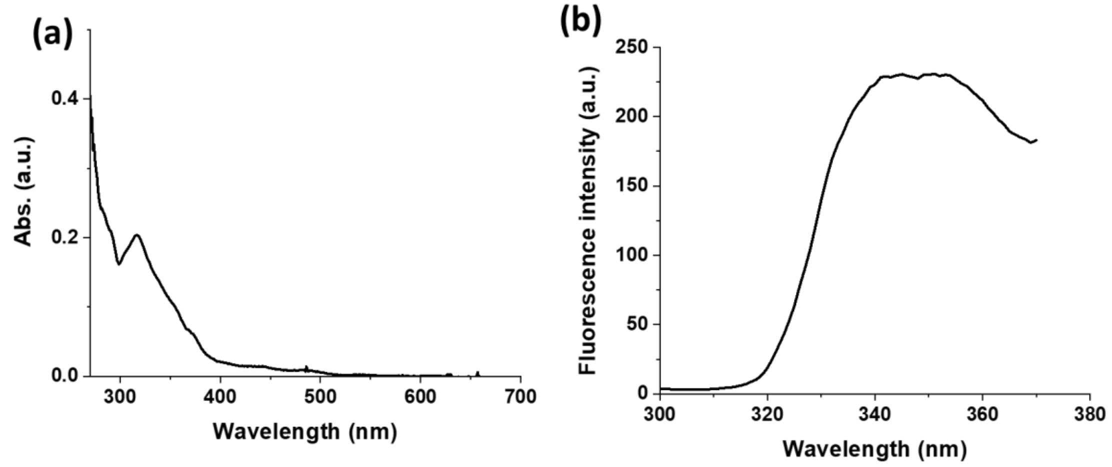

Figure S21. (a) UV absorption and (b) excitation spectra of TPE-APOSS in DMF.

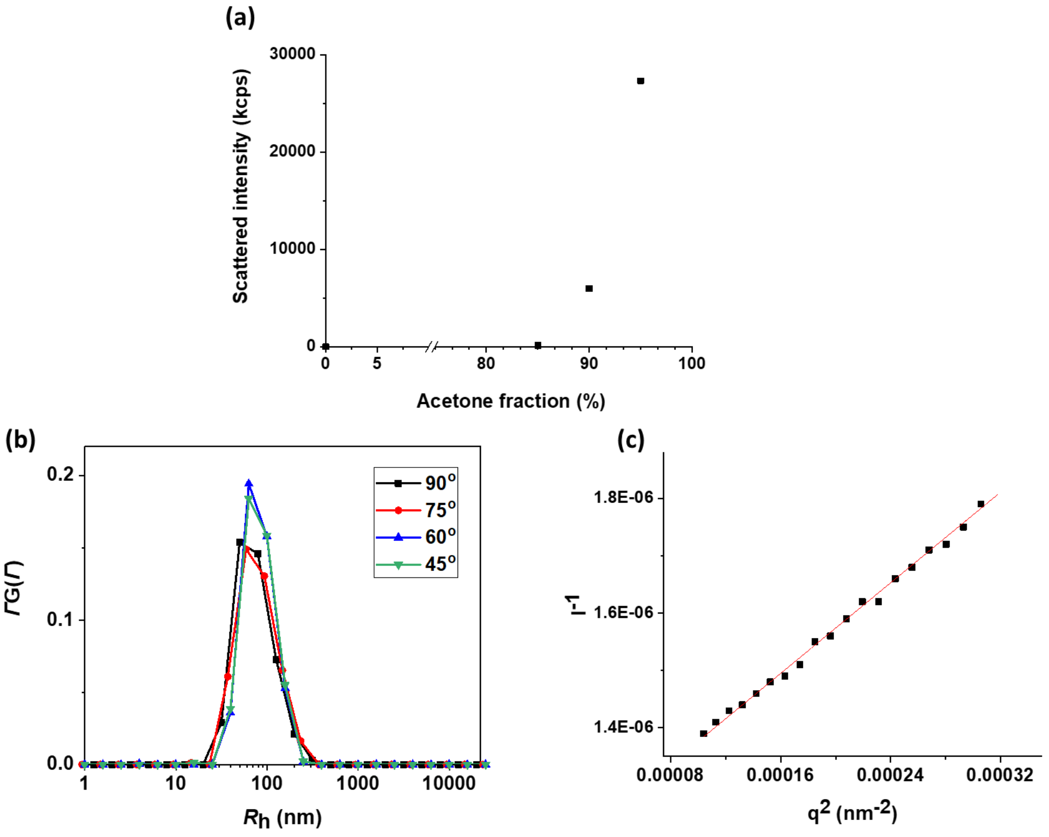

Figure S22. (a) The scattered intensity of 0.3 mg/mL TPE-APOSS in DMF/acetone mixed solvent with various acetone volume fractions. (b) The angular independence and (c) R_g of 0.3mg/mL TPPE-APOSS in 90% Acetone/10% DMF mixed solvent (R_g = 69.8 ± 0.5 nm).

S3. Molecular dynamics simulations.

The structure of TPPE-APOSS molecules were built *via* Materials Studio package[4]. Initial simulation box was prepared through adding 5 molecules of TPPE-APOSS to the mixture solvents of Acetone and DMF, performed via POLYMATIC package.[5] The molecules were negatively charged through deprotonation of carboxylic functional group attached to APOSS in random. Two systems with total partial charge of -15 and -13 were considered where they could be analogous to the experimental cases of 85% and 95% acetone/DMF, respectively. For solvent systems with 85% and 95% acetone/DMF, the simulation box was filled with 8500/1500 and 9500/500molecules of acetone/DMF, respectively. Extra Na^+ cations were added to the simulation box to ensure that the system is charge neutral. The intramolecular and intermolecular interactions were defined through employing OPLSAA forcefield[6] where density functional theory was used for partial charge calculation of TPPE/APOSS molecules. To do so, B3LYP/6-

31++(d,p) functional/basis set was used for calculating CM5 partial charge[7,8] for the TPPE molecule while original OPLSAA partial charges are assigned to DMF and acetone. Molecular dynamics simulations were performed utilizing the LAMMPS software package[9], with periodic boundary conditions applied across all dimensions. The 12Å cutoff was set for Van der Waals interaction and short-range electrostatic interactions while PPPM algorithm with accuracy of 10^{-4} would calculate the long-range electrostatic interactions. After minimizing the initial simulation box, NPT ensemble at T=300K and P=1 atm was applied to the system till the box density reaches equilibrium value. Further simulation was performed in NVT ensemble at the same temperature for about 300 ns. The time step of the simulation was set to 1 fs and dumping frequency performed every 5ps.

To obtain the correlation time for ethylenic dihedral angles in TPPE-APOSS, the autocorrelation function (ACF) is calculated via Equation 1

$$\mathrm{ACF}(\tau) = \frac{\langle \cos\phi_t . \cos\phi_{t+\tau} \rangle}{\langle \cos\phi_t . \cos\phi_t \rangle} \quad (\text{eq. } 1)$$

Where φ is the dihedral angle and τ is the time unit difference of frames with minimum value of 5ps. After calculating autocorrelation function, the corresponding data are fitted with Kohlrausch-Williams-Watts equation[10] for further investigation of relaxation time, shown in Equation 2.

$$\mathrm{ACF}(\tau) = \mathrm{A}\exp\left(-\left(\frac{\tau}{\tau_{\mathrm{KWW}}}\right)^{c}\right) \quad (\text{eq. } 2)$$

From the fitting, parameters of A, c and τ_{KWW} are extracted and total relaxation time τ is calculated by using equation 3 where corresponding values are shown in Table S1.

$$\tau = \int \mathrm{ACF}(\tau)\, d\tau = \frac{\mathrm{A}.\tau_{\mathrm{KWW}}}{c}\Gamma\left(\frac{1}{c}\right) \quad (\text{eq. } 3)$$

Table S1. Fitting parameters for Kohlrausch-Williams-Watts equation for dihedral angle α

	**A**	τ_{KWW} (ns)	c	τ (ns)
**Lower charged TPPE-APOSS assembled in 95% acetone/DMF**	0.958	10.41	0.6244	14.26
**Higher charged TPPE-APOSS assembled in 85% acetone/DMF**	0.961	8.17	0.5993	11.83

References:

[1] Y. Li, K. Guo, H. Su, X. Li, X. Feng, Z. Wang, W. Zhang, S. Zhu, C. Wesdemiotis, S. Z. D. Cheng, *Chem Sci* **2014**, *5*, 1046–1053.

[2] F. Feher, K. Wyndham, R. Baldwin, J. Ziller, J. Lichtenhan, *Chemical Communications* **1999**, 1289–1290.

[3] W. Zhang, Y. Chu, G. Mu, S. A. Eghtesadi, Y. Liu, Z. Zhou, X. Lu, M. A. Kashfipour, R. S. Lillard, K. Yue, T. Liu, S. Z. D. Cheng, *Macromolecules* **2017**, *50*, 5042–5050.

[4] Material Studio™, by Dassault Systèmes BIOVIA, UK (Accelrys®), (License purchased by The University of Akron).

[5] L. J. Abbott, K. E. Hart, C. M. Colina, *Theor Chem Acc* **2013**, *132*, 1–19.

[6] E. K. Watkins, W. L. Jorgensen, *Journal of Physical Chemistry A* **2001**, *105*, 4118–4125.

[7] X. Xu, J. Wu, Z. Lin, A. H. Mokarizadeh, X. H. Li, M. Tsige, S. Z. D. Cheng, T. Liu, *Macromolecules* **2024**, *57*, 955–962.

[8] 1.14* CM1A-LBCC: localized bond-charge corrected CM1A charges for condensed-phase simulations

[9] A. P. Thompson, H. M. Aktulga, R. Berger, D. S. Bolintineanu, W. M. Brown, P. S. Crozier, P. J. in ’t Veld, A. Kohlmeyer, S. G. Moore, T. D. Nguyen, R. Shan, M. J. Stevens, J. Tranchida, C. Trott, S. J. Plimpton, *Comput Phys Commun* **2022**, *271*, 108171.

[10] G. Williams, D. C. Watts, *Transactions of the Faraday Society* **1970**, *66*, 80–85.
